# Environmental and financial cost of surgical-site infection by severity after lower limb vascular surgery

**DOI:** 10.1093/bjsopen/zraf015

**Published:** 2025-05-07

**Authors:** Ross Lathan, Hannah Daysley, Bharadhwaj Ravindhran, Arthur Lim, Joseph Cutteridge, Misha Sidapra, Judith Long, Louise Hitchman, Pedro Beltran-Alvarez, Daniel Carradice, George Smith, Ian Chetter

**Affiliations:** Academic Vascular Surgical Unit, Hull University Teaching Hospital NHS Trust, Hull, UK; Centre for Clinical Sciences, Hull York Medical School, Hull, UK; Academic Vascular Surgical Unit, Hull University Teaching Hospital NHS Trust, Hull, UK; Centre for Clinical Sciences, Hull York Medical School, Hull, UK; Academic Vascular Surgical Unit, Hull University Teaching Hospital NHS Trust, Hull, UK; Centre for Clinical Sciences, Hull York Medical School, Hull, UK; Academic Vascular Surgical Unit, Hull University Teaching Hospital NHS Trust, Hull, UK; Centre for Clinical Sciences, Hull York Medical School, Hull, UK; Academic Vascular Surgical Unit, Hull University Teaching Hospital NHS Trust, Hull, UK; Centre for Clinical Sciences, Hull York Medical School, Hull, UK; Academic Vascular Surgical Unit, Hull University Teaching Hospital NHS Trust, Hull, UK; Centre for Clinical Sciences, Hull York Medical School, Hull, UK; Academic Vascular Surgical Unit, Hull University Teaching Hospital NHS Trust, Hull, UK; Centre for Clinical Sciences, Hull York Medical School, Hull, UK; Academic Vascular Surgical Unit, Hull University Teaching Hospital NHS Trust, Hull, UK; Centre for Clinical Sciences, Hull York Medical School, Hull, UK; Biomedical Institute for Multimorbidity, Centre for Biomedicine, Hull York Medical School, The University of Hull, Hull, UK; Academic Vascular Surgical Unit, Hull University Teaching Hospital NHS Trust, Hull, UK; Centre for Clinical Sciences, Hull York Medical School, Hull, UK; Academic Vascular Surgical Unit, Hull University Teaching Hospital NHS Trust, Hull, UK; Centre for Clinical Sciences, Hull York Medical School, Hull, UK; Academic Vascular Surgical Unit, Hull University Teaching Hospital NHS Trust, Hull, UK; Centre for Clinical Sciences, Hull York Medical School, Hull, UK

## Abstract

**Background:**

There is sparse evidence of the relationship between environmental and financial costs of surgical-site infection. Identifying areas of high-cost burden would enable key targets for clinical interventions to aid in achieving the UK national net zero healthcare system strategies. The aim of this study was to evaluate the environmental and financial costs of surgical-site infection, subclassified by severity of infection.

**Methods:**

This prospective observational study evaluated patients with and without surgical-site infection after a variety of lower limb vascular surgery using National Health Service and Personal and Social Services perspectives. The severity of surgical-site infection was defined using both Centers for Disease Control and Prevention and management-based criteria where patients with mild surgical-site infection required oral antibiotics, patients with moderate surgical-site infection required intravenous antibiotics, and patients with severe surgical-site infection required further surgical interventions.

**Results:**

A total of 99 patients were included, with 22 patients (22.2%) diagnosed with surgical-site infection. The mean(s.d.) environmental cost without surgical-site infection was 10.3(24.3) kgCO_2_e (95% c.i. 4.8 to 15.9) per patient. Emissions increased with surgical-site infection severity, with mild producing 94.6(53.9) kgCO_2_e (95% c.i. 63.5 to 125.8, 918% increase), moderate producing 648(407.6) kgCO_2_e (95% c.i. −0.1 to 1296.6, 6291% increase) and severe producing 2651.4(2217.1) kgCO_2_e (95% c.i. −966.5 to 6347.2, 25 742% increase) per patient. The mean(s.d.) financial cost without surgical-site infection was €73.26(€160.27) (95% c.i. 36.91 to 109.72) that increased with severity, with mild costing €392.25(225.69) (95% c.i. 262.16 to 523.00, 536% increase), moderate costing €9754.46(5059.77) (95% c.i. 1704.65 to 17 820.68, 13 317% increase), and severe costing €37 035.60(32 910.84) (95% c.i. −15 376.07 to 89 447.52, 50 521% increase) per episode of infection (£1 = €1.20 (conversion date 25 October 2024)).

**Conclusion:**

Environmental and financial costs are strongly correlated with surgical-site infection severity and display an exponential increase as severity increases. Overall, surgical-site infection incurs a cost of €15.58 for every kgCO_2_e produced. Environmental discounting should be explored and incorporated into sustainability assessments for robust accounting methodology. Surgical-site infection should be evaluated for severity rather than as a binary outcome for comprehensive assessment.

## Teaser

Surgical-site infection is strongly correlated with both rising financial and environmental costs. Reporting of the severity of infection is imperative for accurate interpretation of intervention effect sizes and health technology assessment.

## Introduction

Surgical-site infection (SSI) is the most common healthcare-associated infection. The incidence of SSI varies by procedure and surgical specialty, and can be as high as 40% in lower limb arterial procedures^1^. For patients, a complication with SSI results in additional healthcare visits, prolonged wound healing, and both physical and psychological distress^2,3^. With severe SSI, patients can also require hospitalization and additional surgical intervention, which can lead to limb loss and mortality in some cases^4,5^. SSI is often evaluated as a binary (present/absent) outcome measure, though it is likely that there is an unequal distribution in infection severity when SSI occurs. Patients treated with a course of oral antibiotics in the community are likely to have a very different experience, and require less resource use, compared with those who are hospitalized or require further surgical intervention to manage their infection.

Aside from this clinical burden, there are other substantial costs associated with SSI. The financial costs of SSI have been estimated to be in the region of €4531-€7324 per infection^6,7^ when considering the National Health Service (NHS) cost perspective, though some estimates extend far above this range of values^8^. The variation in cost estimates is likely due to the large variation in SSI incidence with procedure, specialty, and geography, but may also be related to the significant heterogeneity in infection severity.

The NHS in England is committed to reducing its carbon footprint and delivering a ‘net zero’ service by 2045. Identification of problematic areas with high environmental cost may be key to targeting strategies and clinical interventions toward achieving this goal^9^. The environmental cost of SSI in cardiac surgery has been estimated to be between 5 and 2615 kgCO_2_e per episode^10^. However, this was a secondary outcome of the study and the exact methods were not clear. In addition, severity was not assessed and patients without SSI were not evaluated for comparison.

The aim of this study was to evaluate the environmental and financial costs associated with SSI in patients undergoing lower limb vascular procedures and to explore the relationships between these costs and SSI severity.

## Methods

### Study design

This prospective observational cohort study mapped financial costs (in €) and environmental costs (in kgCO_2_e) of patients with and without SSI after lower limb vascular surgery. All patients provided written consent as part of an ongoing randomized clinical trial (RCT) (NCT02992951). Ethical approval for this trial was obtained (16/LO/2135) from the London—Harrow Research Ethics Committee and study conduct was in accordance with the Declaration of Helsinki (2013)^11^. Eligibility criteria followed the parent trial including all adult patients with mental capacity undergoing clean or clean-contaminated lower limb vascular surgery for critical limb threatening ischaemia (with or without tissue loss) or open venous procedures. Patients on concurrent antibiotics at the time of screening for conditions not related to the index procedure were not eligible for enrolment. Patients were recruited between 13 May 2022 and 9 October 2023, in a tertiary vascular centre in the UK.

A process analysis rich approach to carbon accounting modelling for a hybrid life cycle analysis (LCA) was performed. International Standards ISO 14060:2006 for quantification and reporting of greenhouse gases were followed^12^. Costs were evaluated using NHS and Personal and Social Services (PSS) perspectives. Financial costs are additionally converted into Euros for comparison £1 = €1.20 (conversion date 25 October 24). Healthcare resource use was evaluated to base environmental and financial costs.

The environmental and financial costs from each index procedure and routine post-procedure stay (defined by operative clinician) were not included as costs should be equivalent until this point. After the planned discharge date all emissions and financial costs were mapped, i.e. if discharge was delayed or from the point of discharge. Instances of SSI that developed during the initial inpatient stay were evaluated. The environmental and financial costs were evaluated until 3 months post-procedure. Follow-up was conducted using a ‘remote first’ model, whereby all patients had a planned remote appointment 30 days post-surgery consisting of wound assessment via submitted wound images combined with a modified Bluebelle Wound Healing Questionnaire^13^. If a wound-related problem was identified remotely, patients were then seen face-to-face. Reviews were conducted earlier if patients reported concerns to the clinical team. SSI was diagnosed using the Centers for Disease Control and Prevention (CDC) criteria^14^. Two comparative SSI severity classifications were used: CDC criteria^14^ (superficial incisional, deep incisional, and organ/space SSI) and management-based criteria (mild, managed with oral antibiotics; moderate, managed with intravenous antibiotics; and severe, required surgical intervention in addition to antibiotics).

### Outcomes

#### Primary outcome

The primary outcome was the environmental cost (kgCO_2_e) of mild, moderate, and severe SSI compared with no SSI.

#### Secondary outcomes

The secondary outcomes were: the financial cost (€) of mild, moderate, and severe SSI; the distribution of emissions (kgCO_2_e) across healthcare resource use activity areas; the distribution of financial cost (€) across healthcare resource use activity areas; and the financial-environment cost (€/kgCO_2_e) for mild, moderate, and severe SSI.

### Data collection

All consumable and reusable items and packaging were weighed using a Model Scout Pro (SPU123) Electronic Balance for items less than or equal to 120 g and Marsden medical weighing scales (DS-673SS) for items greater than 120 g for evaluation of production and waste emissions values. All items included are shown in the *Supplementary material*.

#### Follow-up review

The same process for mapping remote and face-to-face reviews was used. Resources evaluated during follow-up reviews included time taken (minutes), staff involved (profession and grade), consumables used during review (such as dressings, sodium chloride for sterilization, gauze, gloves, paper bed roll, and disposable aprons), estimated water use for hand-washing (at 0.0051 m^3^), room size (286 × 460 × 220 cm), and lighting (bulb type and quantity). Data on time, room size, and lighting were combined with energy tariffs and emissions factors^15^ for heating and electricity use. These energy data were also used to evaluate environmental and financial costs from computer use. Consumables and their packaging were evaluated for environmental cost for their production, use, and disposal. Each item was weighed and emissions factors for production were used based upon item material and number used. Disposal was evaluated based upon whether items underwent high or low energy incineration and associated emissions factors. NHS supply chain data were used for consumable item costs^16^. Costs associated with patient travel were included, using distance travelled from home to clinic postcode, parking costs, and mode of travel.

#### Other reviews

Reviews were evaluated based upon 10 min for general practitioner review and 30-min appointments for all other reviews. National tariff data were taken from Personal Social Services Research Unit (PSSRU)^17^ and emissions factors for staff in person and remote consultation factors from the Sustainable Development Unit (SDU)^18^.

#### Imaging

The National Institute for Health and Care Research interactive costing tool for investigation and intervention tariffs provided radiological investigation financial costs^19^ and emissions factors per scan were taken from previously published models^20^.

#### Admission

Additional low-dependency and high-dependency ward days were recorded for each patient. Financial costs were sourced using models from 2016/2017 NHS costs inflated to 2022/2023 prices^21^. Emissions factors for each were taken from SDU data^18^.

#### Pharmaceuticals

All medications were evaluated for production using cost and pharmaceutical emissions factors. Packaging was appraised for production and disposal using Department for Environment, Food, and Rural Affairs (DEFRA) emissions factors, material, and weight^15^. British National Formulary (BNF) formulated the basis for pharmaceutical cost calculations^22^.

#### Consumable items

All consumable items were evaluated for composition material, including packaging, with each part weighed. DEFRA emissions factors were then applied to estimate emissions for production and disposal^15^. NHS supply chain data provided individual item financial costs^16^. In addition to disposable gloves, aprons, towels, dressings, and wound cleaning equipment, data were collected on blood and microbiological samples and cannulation items.

#### Procedures

For all surgical procedures, data on consumable items, pharmaceuticals, and staff time were evaluated for environmental and financial costs as described. Additionally, reusable items underwent sterilization processes in line with local practices. Post-procedure items underwent 90°C thermal disinfection using 45-min cycles with autoclaving at 144°C for 3.5 min. Instrument sets were weighed and calculated for production using DEFRA emissions factors over the estimated lifetime usage (2040 uses)^15^. Energy tariff data were then used to estimate emissions for reusable sterilization processes. Data on anaesthetic gases were derived from operating time and emissions factors provided by the Association of Anaesthetists Anaesthetic Gases Calculator^23^.

#### Data analysis

Data were collected and entered into SPSS^®^ (IBM, Armonk, NY, USA; version 28) and a two-sided *P* < 0.050 was considered statistically significant. Descriptive statistics are presented as *n* (%), mean(s.d.), or median (interquartile range) as appropriate. Groups were compared using the Mann–Whitney *U* test for binary SSI outcomes and the one-way ANOVA test with Tukey post-hoc analysis for SSI severity. SSI severity and financial and environmental costs were plotted on scatter graphs with lines of best fit plotted to establish whether relationships were linear or monotonic. Correlation was then assessed between SSI severity and financial or environmental costs using the Spearman rank correlation coefficient. Pearson correlation was used to assess the relationship between financial and environmental costs. Calculations for carbon offsetting value in trees planted were based upon the kgCO_2_e sequestered over 1 year by a 10-year-old, 5-m tall, 25-cm diameter tree with a dry weight of 155.6 kg^24^.

Sensitivity analyses were conducted for financial and environmental costs by varying the mean and median antibiotic days by the 95% confidence interval and interquartile range.

## Results

A total of 105 patients were enrolled into the study with a high proportion being elderly, male, and with a smoking history (*Table 1*). There were two cancelled procedures, three perioperative deaths (two cardiac events and one pulmonary embolus), and 1 patient lost to follow-up, leaving 99 patients for analysis. An SSI was diagnosed in 22 patients (SSI rate of 22.2%). Using the management-based SSI severity criteria, 14 patients (63.3%) had mild SSI, 4 patients (18.2%) had moderate SSI, and 4 patients (18.2%) had severe SSI. Using the CDC SSI severity criteria, 11 patients (50.0%) had superficial SSI, 8 patients (36.4%) had deep SSI, and 3 patients (13.6%) had organ/space SSI.

**Table 1:** Baseline patient characteristics

	No SSI (*n* = 77)	SSI (*n* = 22)	*P*
Age (years), mean(s.d.)	67.66(11.56)	65.18(11.75)	0.797
**Sex**			
Male	60 (77.9)	13 (59.1)	
Female	17 (22.1)	9 (40.9)	0.077
BMI (kg/m^2^), mean(s.d.)	25.17(4.13)	28.53(6.41)	0.424
**Smoking history**			
Non-smoker	10 (13.0)	2 (9.1)	
Ex-smoker	42 (54.5)	10 (45.5)	
Current smoker	25 (32.5)	10 (45.5)	0.522
**Diabetes**			
None	42 (54.5)	13 (59.1)	
Diet controlled	9 (11.7)	1 (4.5)	
Non-insulin dependent	17 (22.1)	2 (9.1)	
Insulin dependent	9 (11.7)	6 (27.3)	0.159
**Previous CVA/TIA**			
No	65 (84.4)	21 (95.5)	0.209
Yes	12 (15.6)	1 (4.5)	
**Hypertension**			
No	30 (39.0)	8 (36.4)	
Yes	47 (61.0)	14 (63.6)	0.741
**IHD**			
No	41 (53.2)	14 (63.6)	
Yes	36 (46.8)	8 (36.4)	0.387
**COPD**			
No	59 (76.6)	11 (50.0)	
Yes	18 (23.4)	11 (50.0)	0.016
**CKD**			
No	59 (76.6)	17 (77.3)	
Yes	18 (23.4)	5 (22.7)	0.949
**Procedure**			
Common femoral endarterectomy	13 (16.9)	6 (27.3)	
Femoro-popliteal bypass	37 (48.1)	6 (27.3)	
Femoro-distal bypass	11 (14.3)	6 (27.3)	
Femoro-femoral crossover	1 (1.3)	2 (9.1)	
Aorto-bifemoral bypass	10 (13.0)	1 (4.5)	
Major lower limb amputation	4 (5.2)	0 (0.0)	
Femoral or popliteal embolectomy	0 (0.0)	1 (4.5)	
Saphenofemoral junction ligation	1 (1.3)	0 (0.0)	0.055
**Redo procedure**			
No	73 (94.8)	21 (95.5)	
Yes	4 (5.2)	1 (4.5)	0.902
**Emergency procedure**			
No	76 (98.7)	21 (95.5)	
Yes	1 (1.3)	1 (4.5)	0.34
**Prosthetic**			
No	51 (66.2)	19 (86.4)	
Yes	26 (33.8)	3 (13.6)	0.067

Values are *n* (%) unless otherwise indicated. SSI, surgical-site infection; CVA, cerebrovascular accident, TIA, transient ischaemic attack; IHD, ischaemic heart disease; COPD, chronic obstructive pulmonary disease; CKD, chronic kidney disease.

### Emissions associated with surgical-site infection

Overall, the mean(s.d.) emissions produced by patients without SSI was 10.3(24.3) kgCO_2_e (95% c.i. 4.8 to 15.9, 0.4(0.9) trees) per patient. For patients who developed SSI, the mean(s.d.) carbon emissions was over 60-fold higher at 643.8(1276.4) kgCO_2_e (95% c.i. 110.4 to 1177.2, 22.6(44.8) trees) per patient (mean difference of 633.5 kgCO_2_e (95% c.i. 348.3 to 918.6), *t*(97) = −4.409, *P* < 0.001).

**Fig. 1:**
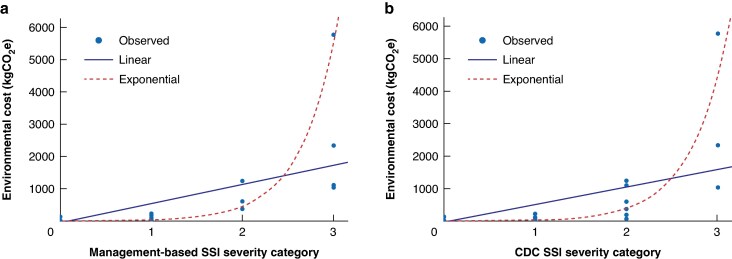
Scatter plots of environmental cost (kgCO_2_e) by surgical-site infection severity category **a** Scatter plot of environmental cost by management-based surgical-site infection severity category: R^2^ linear = 0.462 and adjusted R^2^ exponential = 0.677 (with outlier value removed: R^2^ linear = 0.663 and adjusted R^2^ exponential = 0.645). **b** Scatter plot of environmental cost by Centers for Disease Control and Prevention surgical-site infection severity category: R^2^ linear = 0.412, adjusted R^2^ exponential = 0.675 (with outlier value removed: R^2^ linear = 0.560 and adjusted R^2^ exponential = 0.643). SSI, surgical-site infection; CDC, Centers for Disease Control and Prevention.

Mean(s.d.) emissions increased in line with SSI severity, with mild SSI producing 94.6(53.9) kgCO_2_e (95% c.i. 63.5 to 125.8, 3.3(1.9) trees), moderate SSI producing 648(407.6) kgCO_2_e (95% c.i. −0.1 to 1296.6, 22.7(14.3) trees), and severe SSI producing 2651.4(2217.1) kgCO_2_e (95% c.i. −966.5 to 6347.2, 93.0(77.8) trees) per patient. The difference between groups was significant (*F*(3,95) = 53.298, *P*  *<* 0.001). A Tukey post-hoc test revealed a significant difference in environmental cost between patients with moderate SSI and no infection (*P*  *=* 0.013) and between patients with severe SSI and all other groups (no infection, *P*  *<* 0.001; mild, *P*  *<* 0.001; and moderate, *P*  *<* 0.001) (*Table 2*). There was a moderately positive association between SSI severity and rising carbon emissions (*rs* = 0.698, *P*  *<* 0.001). A scatter plot of environmental cost by SSI severity category suggested a strong exponential relationship (adjusted R^2^ = 0.677, R^2^ linear = 0.462) and marginally favoured a linear relationship with extreme outliers removed (adjusted R^2^ = 0.645, R^2^ linear = 0.663) (*Fig. 1a*).

**Table 2:** Tukey post-hoc analysis of inter-group significance in emission produced by no infection, and both surgical-site infection severity scores (Centers for Disease Control and Prevention and management-based)

Criteria (CDC)	Comparison SSI severity		Management-based SSI severity		CDC SSI severity	
			Mean difference (95% c.i.), kgCO_2_e	*P*	Mean difference (95% c.i.), kgCO_2_e	*P*
No infection	0	1	−84.3 (−389.5,220.9)	0.888	−78.3 (−394.8,238.1)	0.916
		2	−637.8 (−1176.5,−99.2)	0.013	−495.4 (−860.1,−130.8)	0.003
		3	−2551.1 (−3089.8,−2012.4)	<0.001	−3037.0 (−3614.7,−2459.3)	<0.001
Mild (superficial)	1	0	84.3 (−220.9,389.5)	0.888	78.3 (−238.1,394.8)	0.916
		2	−553.5 (−1149.1,42.0)	0.078	−417.1 (−873.2,39.0)	0.086
		3	−2466.8 (−3062.3,−1871.3)	<0.001	−2958.6 (−3598.0,−2319.2)	<0.001
Moderate (deep)	2	0	637.8 (99.2,1176.5)	0.013	495.4 (130.8,860.1)	0.003
		1	553.5 (−42.0,1149.1)	0.078	417.1 (−39.0,873.2)	0.086
		3	−1913.3 (−2656.0,−1170.5)	<0.001	−2541.5 (−3206.1,−1877.0)	<0.001
Severe (organ/space)	3	0	2551.1 (2012.4,3089.8)	<0.001	3037.0 (2459.3,3614.7)	<0.001
		1	2466.8 (1871.3,3062.3)	<0.001	2958.6 (2319.2,3598.0)	<0.001
		2	1913.3 (1170.5,2656.0)	<0.001	2541.5 (1877.0,3206.1)	<0.001

CDC, Centers for Disease Control and Prevention; SSI, surgical-site infection.

Comparatively, mean(s.d.) emissions also increased with CDC SSI severity, whereby management of superficial SSI resulted in 88.7(53.2) kgCO_2_e (95% c.i. 53.0 to 124.4, 3.1(1.9) trees), management of deep SSI resulted in 505.8(447.1) kgCO_2_e (95% c.i. 132.0 to 879.5, 17.7(15.7) trees), and management of organ/space SSI resulted in 3047.3(2440.7) kgCO_2_e (95% c.i. −3015.8 to 9110.4, 106(85.3) trees). The difference between groups was significant (*F*(3,95) = 65.618, *P*  *<* 0.001) and a Tukey post-hoc test identified significant differences between deep SSI and no infection (*P*  *=* 0.003) and between organ/space SSI and all other groups (no infection, *P*  *<* 0.001; superficial SSI, *P*  *<* 0.001; and deep SSI, *P*  *<* 0.001) (*Table 2*). CDC SSI severity was moderately correlated with raised emissions cost (*rs* = 0.697, *P*  *<* 0.001). A scatter plot of environmental cost by SSI severity category suggested a strong exponential relationship (adjusted R^2^ = 0.675, R^2^ linear = 0.412), which was maintained with the extreme outlier removed (adjusted R^2^ = 0.643, R^2^ linear = 0.560) (*Fig. 1b*). There was a very strong positive association between SSI scoring using CDC and management-based methods (*rs* = 0.965, *P*  *<* 0.001).

### Distribution of emissions

Overall, 796.3 kgCO_2_e (range 1.2–134.8, 77 patients, 5.3%) was produced when managing patients without infection compared with 14 163.6 kgCO_2_e (range 33.1–5765.2, 22 patients, 94.7%) when managing patients with SSI. For management-based SSI severity, 1325.1 kgCO_2_e (range 33.1–227.1, 14 patients, 8.9%) was produced when managing patients with mild SSI, 2592.7 kgCO_2_e (range 373.4–1238.2, 4 patients, 17.3%) was produced when managing patients with moderate SSI, and 10 245.8 kgCO_2_e (range 1042.7–5765.2, 4 patients, 68.5%) was produced when managing patients with severe SSI. For CDC SSI severity, treatment of patients with superficial SSI resulted in 975.5 kgCO_2_e (range 60.5–227.1, 11 patients, 6.5%), treatment of patients with deep SSI resulted in 4046.2 kgCO_2_e (range 161.9–1238.2, 8 patients, 27.0%), and treatment of patients with organ/space SSI resulted in 9141.9 kgCO_2_e (range 1688.4–5765.2, 3 patients, 61.1%).

Most emissions were due to readmission (7648.5 kgCO_2_e, 51.1% of all emissions), procedures (2687.6 kgCO_2_e, 18.0% of all emissions), clinical review (2559.8 kgCO_2_e, 17.1% of all emissions), and medications (1705.1 kgCO_2_e, 11.4% of all emissions) (*Table 3*). Ten procedures were performed on the four patients with SSI who required re-intervention: one angioplasty (external iliac and femoro-tibio-peroneal trunk), five wound debridements, one graft thrombectomy, one excision of infected graft with further bypass, one below knee amputation (due to both infection and irretrievable ischaemia), and one combined procedure (wound exploration, graft salvage, angiogram, and drainage of collections). One patient without SSI had a seroma drained in clinic. Across the eight patients who were readmitted, a total of 195 additional bed days were accrued, including 190 days (97.4%) on a low-intensity ward and 5 days (2.6%) on a high-intensity ward.

**Table 3:** Resource use breakdown for all patients and by infection status

	All patients, kgCO_2_e (%)	SSI, kgCO_2_e per patient	No SSI, kgCO_2_e per patient
Procedures	2687.6 (18.0)	122.1	0.0
Review	2559.8 (17.1)	89.5	7.7
Transfer	18.2 (0.1)	0.8	0.0
Admission	7648.5 (51.1)	347.7	0.0
Imaging	64.6 (0.4)	2.6	0.1
Other consumables	72.7 (0.5)	3.3	0.0
Pharmaceuticals	1705.1 (11.4)	77.5	0.0
Dressings	203.3 (1.4)	6.4	0.8
Total	14 959.9 (100.0)	649.8	8.6

SSI, surgical-site infection.

### Financial cost

The mean(s.d.) overall financial cost of SSI was €10 017.66(21 755.48) (95% c.i. 927.56 to 19 124.63) per patient compared with €73.26(160.27) (95% c.i. 36.91 to 109.72) per patient without SSI (mean difference of €9944.41 (95% c.i. 5091.37 to 14 814.18), *t*(97) = −4.063, *P* < 0.001).

**Fig. 2:**
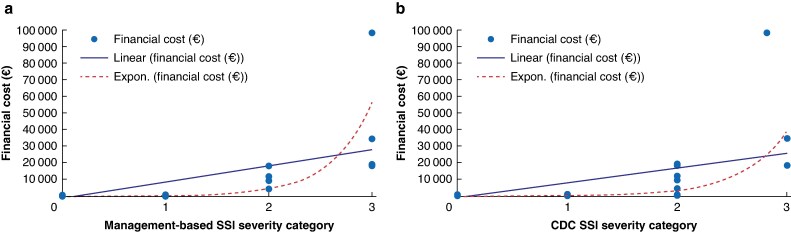
Scatter plots of financial cost (€) by surgical-site infection severity category **a** Scatter plot of financial cost by management-based surgical-site infection severity category: R^2^ linear = 0.441 and adjusted R^2^ exponential = 0.761 (with outlier value removed: R^2^ linear = 0.664 and adjusted R^2^ exponential = 0.730). **b** Scatter plot of financial cost by Centers for Disease Control and Prevention surgical-site infection severity category: R^2^ linear = 0.386 and adjusted R^2^ exponential = 0.724 (with outlier value removed: R^2^ linear = 0.536 and adjusted R^2^ exponential = 0.690). £1 = €1.20 (conversion date 25 October 2024). SSI, surgical-site infection; CDC, Centers for Disease Control and Prevention.

The mean(s.d.) financial cost increased with SSI severity, with mild SSI costing €392.25(225.69) (95% c.i. 262.16 to 523.00), moderate SSI costing €9754.46(5059.77) (95% c.i. 1704.65 to 17 820.68), and severe SSI costing €37 035.60(32 910.84) (95% c.i. −15 376.07 to 89 447.52, €30 004.43(32 910.84)) per episode. The difference in financial cost between severity groups was significant (*F*(3,95) = 51.979, *P*  *<* 0.001); mean differences between mild SSI episodes and no SSI were not significant (*P*  *=* 0.998), but all other comparisons were significant (*P*  *<* 0.050) (*Table 4*). SSI severity was moderately correlated with financial cost (*rs* = 0.669, *P*  *<* 0.001). A scatter plot of financial cost by SSI severity category suggested a strong exponential relationship (adjusted R^2^ = 0.761, R^2^ linear = 0.441) and continued to favour an exponential relationship with extreme outliers removed (adjusted R^2^ = 0.730, R^2^ linear = 0.664) (*Fig. 2a*).

**Table 4:** Tukey post-hoc analysis of inter-group mean difference in financial cost per episode of no infection, mild surgical-site infection, moderate surgical-site infection, and severe surgical-site infection

Criteria (CDC)	Comparison SSI Severity		Management-based SSI severity				CDC SSI Severity			
			Mean difference	95% Confidence interval		*P* value	Mean difference	95% Confidence interval		*P* value
			(€)	Lower bound	Upper bound		(€)	Lower bound	Upper bound	
No infection	0	1	−319.32	−4820.52	4182.00	0.998	−304.26	−5776.01	5167.49	0.999
		2	−9689.40	−17634.36	−1744.32	0.010	−8060.64	−14366.52	−1754.77	0.006
		3	−36962.40	−44907.48	−29017.44	<0.001	−50376.38	−60366.38	−40386.40	<0.001
Mild (Superficial)	1	0	319.32	−4182.00	4820.52	0.998	304.26	−5167.49	5776.01	0.999
		2	−9370.08	−18153.60	−586.56	0.032	−7756.38	−15644.29	131.52	0.056
		3	−36643.20	−45426.72	−27859.56	<0.001	−50072.12	−61129.02	−39015.23	<0.001
Moderate (Deep)	2	0	9689.40	1744.32	17634.36	0.010	8060.64	1754.77	14366.52	0.006
		1	9370.08	586.56	18153.60	0.032	7756.38	−131.52	15644.29	0.056
		3	−27273.00	−38228.04	−16318.08	<0.001	−42315.74	−53808.30	−30823.18	<0.001
Severe (Organ/Space)	3	0	36962.40	29017.44	44907.48	<0.001	50376.38	40386.40	60366.38	<0.001
		1	36643.20	27859.56	45426.72	<0.001	50072.12	39015.23	61129.02	<0.001
		2	27273.00	16318.08	38228.04	<0.001	42315.74	30823.18	53808.30	<0.001

£1 = €1.20 (conversion date 25 October 2024). SSI, surgical-site infection.

Comparatively, mean(s.d.) financial costs increased with CDC SSI severity, whereby management of superficial SSI cost €377.59(234.55) (95% c.i. 218.68 to 535.27), management of deep SSI cost €8127.11(7900.07) (95% c.i. 1523.77 to 14 744.15), and management of organ/space SSI cost €50 407.24(42 172.47) (95% c.i. −45 333.97 to 129 416.80). The difference between groups was significant (*F*(3,95) = 60.514, *P*  *<* 0.001) and a Tukey post-hoc test identified significant differences between deep SSI and no infection (*P*  *=* 0.006) and between organ/space SSI and all other groups (no infection, *P*  *<* 0.001; superficial, *P*  *<* 0.001; and deep, *P*  *<* 0.001) (*Table 4*). CDC SSI severity was moderately correlated with financial cost (*rs* = 0.668, *P*  *<* 0.001). A scatter plot of financial cost by SSI severity category suggested a strong exponential relationship (adjusted R^2^ = 0.724, R^2^ linear = 0.386) and continued to favour an exponential relationship with extreme outliers removed (adjusted R^2^ = 0.690, R^2^ linear = 0.536) (*Fig. 2b*).

**Fig. 3:**
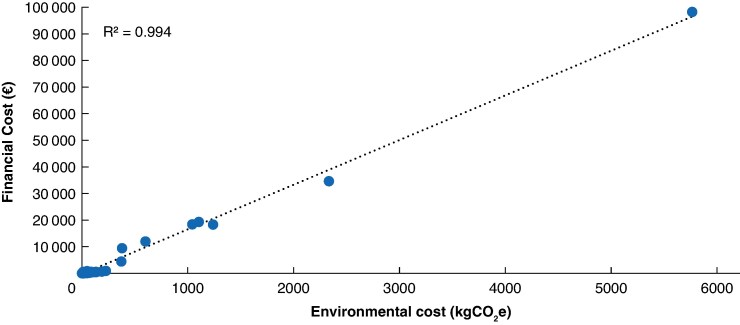
Scatter plot of financial cost (€) by environmental cost (kgCO_2_e) Breakdown is shown by management-based surgical-site infection severity category. R^2^ = 0.994. £1 = €1.20 (conversion date 25 October 2024). SSI, surgical-site infection.

Financial costs per patient were largely attributable to readmission (€119 614.90, 72.4% of all SSI-related costs), followed by procedures (€14 911.40, 7.5% of all SSI-related costs), clinical review (€14 446.38, 7.3% of all SSI-related costs), medications (€12 675.63, 6.4% of all SSI-related costs), and imaging (€11 316.07, 5.7% of all SSI-related costs) (*Table 5*). In patients with SSI, financial costs displayed a linear relationship with environmental costs (*Fig. 3*).

**Table 5:** Financial cost breakdown by resource use category and surgical-site infection status

	All patients (n=99)	%	SSI	No SSI
	€		€/patient	€/patient
Procedures	14923.96	(6.6)	676.72	0.47
Review	14458.55	(6.4)	493.79	46.69
Transfer	270.00	(0.1)	12.28	0.00
Admission	285653.90	(75.8)	7790.56	0.00
Imaging	11325.60	(5.0)	444.32	20.14
Other consumables	239.77	(0.1)	10.34	0.05
Pharmaceuticals	12686.30	(5.6)	576.56	0.02
Dressings	742.80	(0.3)	23.28	3.00
	226030.33	(100.00)	10027.85	70.36

£1 = €1.20 (conversion date 25 October 2024). SSI, surgical-site infection.

### Sensitivity analyses

The mean and median antibiotic days for all SSI (mean 24.75, median 18.75) and each SSI severity category (mild (mean 13.50; median 7.00), moderate (mean 41.00; median 31.50), and severe (mean 47.92; median 50.58)) were varied within their respective 95% confidence intervals for sensitivity analyses for both environmental and financial cost. For all SSI emissions, data were skewed, ranging from 91.86 to 1163.02 kgCO_2_e per episode. Emissions cost estimates rose exponentially with increasing severity, with mild resulting in 59.84–115.68 kgCO_2_e, moderate resulting in 350.28–946.08 kgCO_2_e, and severe resulting in 1054.41–4068.50 kgCO_2_e. All SSI episode financial cost estimates were skewed, ranging from €453.24 to €19 003.55. Episode estimates all rose exponentially with increasing severity, with mild SSI resulting in €174.70–€462.63, moderate resulting in €5419.15–€16 753.08, and severe resulting in €5911.88–€79 364.35. All sensitivity analyses are shown in *Table 6*. Financial cost per emissions produced in each category appeared to be highest for moderate SSI.

**Table 6:** Sensitivity analyses for emissions and financial cost of all surgical-site infections and by surgical-site infection severity category

SSI Category	Method	Antibiotic days	Estimated Emissions Cost (kgCO_2_e)			Estimated Financial Cost (€)			Financial - Emissions Cost Ratio
			Mean	Lower 95% CI*	Upper 95% CI	Mean	Lower 95% CI*	Upper 95% CI	Financial cost (€)/Emissions Cost (kgCO_2_e)
All SSI	Mean	24.75	643.78	124.54	1163.02	10,017.66	927.56	19,124.63	15.58
	Median	18.75	111.67	91.86	550.03	525.40	453.62	11155.20	4.70
Mild SSI	Mean	13.50	94.65	73.61	115.68	392.58	322.14	463.02	4.15
	Median	7.00	65.14	59.84	81.30	264.89	174.70	312.77	4.07
Moderate SSI	Mean	41.00	648.18	350.28	946.08	11095.45	5423.71	16767.19	17.11
	Median	31.50	518.22	354.67	788.37	10425.12	7545.40	13807.57	20.11
Severe SSI	Mean	47.92	2561.45	1054.41	4068.50	42674.03	5916.86	79431.20	16.66
	Median	50.58	1706.50	1092.46	3158.48	26983.37	19238.80	50465.65	15.82

*Interquartile range used for the median estimates. Mean and median additional antibiotic days were used throughout. £1 = €1.20 (conversion date 25 October 24). SSI, surgical-site infection.

## Discussion

In patients with SSI, environmental and financial costs are correlated strongly in a linear relationship (R^2^ = 0.994). There appears to be an exponential increase in financial and environmental cost as SSI severity increases, though this may be influenced by outlying values and warrants further evaluation. Every 1 kgCO_2_e produced by SSI incurs a financial cost of €15.56. Across severity, instances of mild SSI are relatively inexpensive, costing €4.70 per kgCO_2_e; however, costs rise for moderate and severe SSI (€17.10 and €16.64) per kgCO_2_e respectively). Moderate and severe SSI accounted for a small proportion (8 of 22, 36.4%) of infections in this study, but are responsible for most of both the environmental (88.2%) and financial (95.1%) costs. Strategies to prevent SSI may offer substantial environmental and financial benefits, but targeted interventions that might reduce the severity of SSI are likely to have an even more significant benefit with regard to environmental and financial costs. RCTs of interventions to prevent or reduce SSI should ideally report SSI severity to allow for patient impact, as well as environmental and financial costs, to be explored in evaluations. Further to this, sustainable strategies should focus on prevention of disease to provide considerable environmental benefit in addition to considering utilization of green equipment alternatives.

SSI severity classification has often been under-reported in RCTs, which may reflect its complex definition that requires interpretation for each surgical specialty. Both the accepted CDC SSI severity and management-based SSI severity were strong predictors of environmental cost (CDC SSI severity, adjusted R^2^ = 0.675; and management-based SSI severity, adjusted R^2^ = 0.677) and financial costs (CDC SSI severity, adjusted R^2^ = 0.724; and management-based SSI severity, adjusted R^2^ = 0.761) in this study. Further, the CDC and management-based methods were very strongly correlated (*rs* = 0.965). The breakdown of SSI did change, however, with more patients in the deep SSI category (using the CDC SSI severity criteria) than in the moderate SSI category (using the management-based SSI severity criteria). Whilst strong associations were shown, these differences in severity distributions may alter findings and warrant investigation in larger studies.

Readmission was responsible for the largest proportions of carbon emissions (51.1%) and financial costs (75.8%). Prevention of moderate and severe SSI is likely to have the largest impact on healthcare resource use and carbon emissions. Policies to reduce traditionally inpatient hospital treatments, such as outpatient antibiotic therapy services, may offer both financial and environmental cost-efficient approaches. Further development and evaluation of these optimized regional service delivery schemes in specialty areas should be explored. Surgical procedures accounted for 18.0% of emissions and 6.6% of financial costs in this study and sustainable strategies, such as total intravenous anaesthesia, reusable textiles (hats, gowns, drapes, and trolley covers), and condensed instrument trays (though the size of tray needs to be reduced to make an impact), have been suggested to improve environmental cost, though cost evaluation and clinician acceptability would also need to be considered^25–27^.

Carbon emissions related to SSI have not previously been explored. Boundary setting in studies aiming to capture SSI-related emissions risk substantial heterogeneity due to the variable time frame of diagnosis. During previous reviews SSI follow-up varied from 10 to 90 days post-surgery^28^. This study aimed to encompass emissions related to the sequelae of SSI in addition to the initial infection diagnosis, therefore providing a comprehensive understanding of the emissions cost. Financial costs estimations here (mean €10 017.66 per episode) are representative of prior modelling of SSI costs (€4531–€7324), accounting for inflation and the broad perspectives considered in this study^6,7^.

The environmental cost of SSI will vary year on year as national emissions factors change with rising or falling progress towards net zero targets. Common practice in economic analyses is to apply inflation on historical prices to account for rising financial costs. Similarly, sustainable discounting must be applied to environmental analyses of healthcare activity to account for the changing environmental cost landscape over time. Currently, no such accepted application exists and further workstreams should endeavour to ascertain such values for robust carbon accounting methodologies. Further, ‘dual discounting’ may lead to an alternative financial discounting rate for economic analyses, when factoring in the net long-term positive benefits of sustainable outcomes^29^. Exploration of the linear relationship between environmental and financial cost (and non-linear with SSI severity) in wider contexts over time may yield such answers. Without accounting for environmental discounting, incorporating sustainable outcomes into health technology assessment will risk erroneous appraisal of novel interventions. Hybrid life cycle assessments were utilized here, though there is currently a paucity of methodology on reporting environmental costs in studies. Further research is needed to standardize reporting in development of criteria to be included in such workstreams. The findings here will be compared with a variety of methodologies to ensure accuracy of reporting including financial and weight-based calculations in addition to carbon accounting software using life-cycle assessment that incurs financial costs.

This study has several limitations. First, the study was conducted in a single UK tertiary vascular centre within the NHS, limiting the generalizability of the findings. Additionally, the small sample size requires corroboration on a larger scale for reliability and external validation. Further evidence from wider UK contexts would substantiate the current environmental and financial valuation of SSI and evidence from non-UK settings could be used for holistic comparison. Follow-up was conducted using a novel hybrid method, that is a ‘remote first’ approach comprising a single planned remote assessment with an in-person review when required. Current follow-up practices within UK vascular surgery vary from face-to-face reviews to remote assessments, as well as combinations based upon clinical experience and risk stratification, which will vary the cost calculations at each site^30^. Alternatively, serial remote assessments have been proposed, which are a promising concept with additional patient benefits, though also provide an added variable to these calculations^10,31^. The ‘remote first’ follow-up system presented is the result of environmental and financial cost modelling within several follow-up strategies and therefore presents the most accurate and representative values available in the literature for post-surgical follow-up. These models will be published separately. Additionally, the geographical location of a surgical centre and the associated variable of distance of patients from the centre will all factor into the diverse costs. Data were collected from general practitioner records where possible on healthcare resource use in the community; however, it is likely that the data captured are not comprehensive with regard to all of the resources used and under-represent the true values for both patients with and without SSI. Sensitivity analyses were conducted using the mean and median additional lengths of stay, providing a likely range of values for environmental and financial costs, considering the variables unable to be accounted for in this analysis.

SSI complicates the patient journey and experience, but also results in substantial environmental and financial costs. Readmission and surgical procedures account for most of the resource use for SSI and the subsequent costs. These factors all vary significantly as the severity of SSI increases, suggesting that future reporting should ideally include an indication of severity rather than simply the presence of SSI. One severe infection has the equivalent environmental and financial cost of 28 and 95 mild infections respectively. Further research is needed to substantiate these data on a wider scale and establish ongoing environmental cost discounting rates as the NHS progresses into a greener future.

## Supplementary Material

zraf015_Supplementary_Data

## Data Availability

All data are available within the manuscript and the *Supplementary material*.
