# Supplementary material for: Environmental and financial cost of surgical-site infection by severity after lower limb vascular surgery
Source: BJS Open. 2025 May 7;9(3):zraf015. doi: 10.1093/bjsopen/zraf015 (PMC12056366; doi:10.1093/bjsopen/zraf015)
Supplement: zraf015_Supplementary_Data [file zraf015_supplementary_data.docx]

Supplementary Material

Table of Contents

[Resource Use for Patients With SSI 2](#_Toc169428833)

[Review 2](#_Toc169428834)

[Transfer 2](#_Toc169428835)

[Admission 3](#_Toc169428836)

[Imaging 3](#_Toc169428837)

[Other Consumables 3](#_Toc169428838)

[Pharmaceuticals 4](#_Toc169428839)

[Procedures 7](#_Toc169428840)

[Dressings 15](#_Toc169428841)

[Resource Use for Patients Without SSI 18](#_Toc169428842)

[Review 18](#_Toc169428843)

[Imaging 18](#_Toc169428844)

[Other Consumables 18](#_Toc169428845)

[Pharmaceuticals 18](#_Toc169428846)

[Procedures 18](#_Toc169428847)

[Dressings 19](#_Toc169428848)

# Resource Use for Patients With SSI

| Review | | | | | | | | | | | | | | | |
| --- | --- | --- | --- | --- | --- | --- | --- | --- | --- | --- | --- | --- | --- | --- | --- |
| Product | Component | Material | Weight (kg) | Cost (£) | Number of uses | Number of scenarios per use | Emissions factor | Waste stream Emission Factor | Production | Waste | Number | Production total | Waste total | Total emissions | Total Cost (£) |
| Nurse face-to-face review | Review |  |  | 28.50 |  |  | 0.3100 |  | 8.8350 |  | 89 | 786.3150 | 0.0000 | 786.3150 | 2536.50 |
| Nurse Telephone | Review |  |  | 13.17 |  |  | 0.1550 |  | 2.0408 |  | 3 | 6.1225 | 0.0000 | 6.1225 | 39.50 |
| Vascular Telephone Review | Review |  |  | 35.75 |  |  | 0.1550 |  | 5.5413 |  | 1 | 5.5413 | 0.0000 | 5.5413 | 35.75 |
| ID telephone review | Review |  |  | 56.50 |  |  | 0.1550 |  | 8.7575 |  | 2 | 17.5150 |  | 17.5150 | 113.00 |
| ID face-to-face review | Review |  |  | 56.50 |  |  | 0.3100 |  | 17.5150 |  | 1 | 17.5150 |  | 17.5150 | 56.50 |
| ID face-to-face OPAT nurse review | Review |  |  | 26.50 |  |  | 0.3100 |  | 8.2150 |  | 1 | 8.2150 |  | 8.2150 | 26.50 |
| ED review | Review |  |  | 53.00 |  |  | 0.3100 |  | 13.8000 |  | 8 | 110.4000 | 0.0000 | 110.4000 | 424.00 |
| Vascular face-to-face clinic | Review |  |  | 55.50 |  |  | 0.3100 |  | 17.2050 |  | 5 | 86.0250 | 0.0000 | 86.0250 | 277.50 |
| GP review telephone | Review |  |  | 41.13 |  |  | 0.1550 |  | 6.3752 |  | 3 | 19.1255 | 0.0000 | 19.1255 | 123.39 |
| GP Precription | Review |  |  | 29.00 |  |  |  |  |  |  | 61 |  |  |  | 1769.00 |

| Transfer | | | | | | | | | | | | | | | |
| --- | --- | --- | --- | --- | --- | --- | --- | --- | --- | --- | --- | --- | --- | --- | --- |
| Product | Component | Material | Weight (kg) | Cost (£) | Number of uses | Number of scenarios per use | Emissions factor | Waste stream Emission Factor | Production | Waste | Number | Production total | Waste total | Total emissions | Total Cost (£) |
| Transfer | Review |  |  | 225.00 |  |  | 0.2747 |  | 18.2368 |  | 1 | 18.2368 |  | 18.2368 | 225.00 |

| Admission | | | | | | | | | | | | | | | |
| --- | --- | --- | --- | --- | --- | --- | --- | --- | --- | --- | --- | --- | --- | --- | --- |
| Product | Component | Material | Weight (kg) | Cost (£) | Number of uses | Number of scenarios per use | Emissions factor | Waste stream Emission Factor | Production | Waste | Number | Production total | Waste total | Total emissions | Total Cost (£) |
| Low-intensity ward days | Review |  |  | 586.89 | 1 | 1 | 37.9000 |  | 37.9000 |  | 190 | 7201.0000 |  | 7201.0000 | 111509.10 |
| High-intensity ward days | Review |  |  | 1621.16 | 1 | 1 | 89.5000 |  | 89.5000 |  | 5 | 447.5000 |  | 447.5000 | 8105.80 |

| Imaging | | | | | | | | | | | | | | | |
| --- | --- | --- | --- | --- | --- | --- | --- | --- | --- | --- | --- | --- | --- | --- | --- |
| Product | Component | Material | Weight (kg) | Cost (£) | Number of uses | Number of scenarios per use | Emissions factor | Waste stream Emission Factor | Production | Waste | Number | Production total | Waste total | Total emissions | Total Cost (£) |
| CXR | Review |  |  | 63.00 |  |  | 0.3100 |  | 0.7300 |  | 11 | 8.0300 |  | 8.0300 | 693.00 |
| MSK XR | Review |  |  | 126.00 |  |  | 0.3100 |  | 0.7300 |  | 7 | 5.1100 |  | 5.1100 | 882.00 |
| CT Head | Review |  |  | 174.00 |  |  | 0.3100 |  | 4.0000 |  | 2 | 8.0000 |  | 8.0000 | 348.00 |
| CT angiogram | Review |  |  | 361.00 |  |  | 0.3100 |  | 2.6100 |  | 10 | 26.1000 |  | 26.1000 | 3610.00 |
| USS | Review |  |  | 95.00 |  |  | 0.3100 |  | 0.5300 |  | 7 | 3.7100 |  | 3.7100 | 665.00 |
| CTPA | Review |  |  | 361.00 |  |  | 0.3100 |  | 2.6100 |  | 1 | 2.6100 |  | 2.6100 | 361.00 |
| PET CT | Review |  |  | 1587.00 |  |  | 0.3100 |  | 2.6100 |  | 1 | 2.6100 |  | 2.6100 | 1587.00 |

| Other Consumables | | | | | | | | | | | | | | | |
| --- | --- | --- | --- | --- | --- | --- | --- | --- | --- | --- | --- | --- | --- | --- | --- |
| Product | Component | Material | Weight (kg) | Cost (£) | Number of uses | Number of scenarios per use | Emissions factor | Waste stream Emission Factor | Production | Waste | Number | Production total | Waste total | Total emissions | Total Cost (£) |
| Foley catheter | Product | Rubber (silicone) | 0.0016 | 3.09 | 1 | 1 | 3.1800 | 1.0740 | 0.0051 | 0.0017 | 3 | 0.0153 | 0.0052 | 0.0204 | 9.27 |
|  | Packaging | LDPE | 0.0023 |  | 1 | 1 | 2.6010 | 0.1720 | 0.0060 | 0.0004 | 3 | 0.0179 | 0.0012 | 0.0191 | 0.00 |
|  | Packaging | Paper | 0.0010 |  | 1 | 1 | 0.9190 | 0.1720 | 0.0009 | 0.0002 | 3 | 0.0028 | 0.0005 | 0.0033 | 0.00 |
| Syringe 20ml | Product | LDPE | 0.0129 | 0.11 | 1 | 1 | 0.3000 | 1.0740 | 0.0330 | 0.0139 | 1 | 0.1650 | 0.0693 | 0.2343 | 0.55 |
|  | Packaging | Paper | 0.0004 |  | 1 | 1 | 0.9190 | 0.1720 | 0.0004 | 0.0001 | 1 | 0.0018 | 0.0003 | 0.0022 | 0.00 |
|  | Packaging | LDPE | 0.0007 |  | 1 | 1 | 2.6000 | 0.1720 | 0.0018 | 0.0001 | 1 | 0.0091 | 0.0006 | 0.0097 | 0.00 |
| Cannula | Product | stainless steel | 0.0610 | 0.71 | 1 | 1 | 0.3000 | 1.0740 | 0.2130 | 0.0655 | 13 | 2.7690 | 0.8517 | 3.6207 | 9.23 |
|  | Packaging | LDPE | 0.0050 |  | 1 | 1 | 2.6006 | 0.1720 | 0.0130 | 0.0009 | 13 | 0.1690 | 0.0112 | 0.1802 | 0.00 |
|  | Packaging | Paper | 0.0040 |  | 1 | 1 | 0.9190 | 0.1720 | 0.0037 | 0.0007 | 13 | 0.0478 | 0.0089 | 0.0567 | 0.00 |
| Bionector | Product | Polyurethane | 0.0100 | 0.51 | 1 | 1 | 0.3000 | 1.0740 | 0.1530 | 0.0107 | 13 | 1.9890 | 0.1396 | 2.1286 | 6.63 |
|  | Packaging | LDPE | 0.0030 |  | 1 | 1 | 2.6006 | 0.1720 | 0.0078 | 0.0005 | 13 | 0.1014 | 0.0067 | 0.1081 | 0.00 |
|  | Packaging | Paper | 0.0020 |  | 1 | 1 | 0.9190 | 0.1720 | 0.0018 | 0.0003 | 13 | 0.0239 | 0.0045 | 0.0284 | 0.00 |
| Tegaderm | Product | LDPE | 0.0100 | 0.14 | 1 | 1 | 1.5400 | 1.0740 | 0.2156 | 0.0107 | 16 | 3.4496 | 0.1718 | 3.6214 | 2.24 |
|  | Packaging | LDPE | 0.0030 |  | 1 | 1 | 2.6006 | 0.1720 | 0.0078 | 0.0005 | 16 | 0.1248 | 0.0083 | 0.1331 | 0.00 |
| 0.9% NaCL 5ml Flush | Product | Pharmaceuticals | 0.0180 | 0.62 | 1 | 1 | 0.3000 | 1.0740 | 0.1860 | 0.0193 | 13 | 2.4180 | 0.2513 | 2.6693 | 8.06 |
|  | Packaging | LDPE | 0.0030 |  | 1 | 1 | 2.6006 | 0.1720 | 0.0078 | 0.0005 | 13 | 0.1014 | 0.0067 | 0.1081 | 0.00 |
| 2% chlorhexadine in 70% alcohol skin wipe | Product | Polypropylene | 0.0014 | 0.02 | 1 | 1 | 0.3000 | 1.0740 | 0.0060 | 0.0015 | 13 | 0.0780 | 0.0195 | 0.0975 | 0.26 |
|  | Packaging | Aluminium | 0.0010 |  | 1 | 1 | 9.1220 | 0.1720 | 0.0091 | 0.0002 | 13 | 0.1186 | 0.0022 | 0.1208 | 0.00 |
| Tourniquet | Product | Silicone | 0.0075 | 0.06 | 1 | 1 | 0.3000 | 1.0740 | 0.0180 | 0.0081 | 13 | 0.2340 | 0.1047 | 0.3387 | 0.78 |
| MCS | Product | LDPE | 0.0130 | 0.11 | 1 | 1 | 0.3000 | 1.0740 | 0.0330 | 0.0140 | 52 | 1.7160 | 0.7260 | 2.4420 | 5.72 |
|  | Packaging | LDPE | 0.0030 |  | 1 | 1 | 2.6006 | 0.1720 | 0.0078 | 0.0005 | 52 | 0.4057 | 0.0268 | 0.4325 | 0.00 |
| EDTA tube (FBC) | Product | Polypropylene | 0.0080 | 0.08 | 1 | 1 | 0.3000 | 1.0740 | 0.0240 | 0.0086 | 84 | 2.0160 | 0.7217 | 2.7377 | 6.72 |
| SST (Biochemistry) | Product | Polypropylene | 0.1000 | 0.33 | 1 | 1 | 0.3000 | 1.0740 | 0.0990 | 0.1074 | 87 | 8.6130 | 9.3438 | 17.9568 | 28.71 |
| Anticoagulant EDTA (G&S) | Product | Polypropylene | 0.0200 | 0.07 | 1 | 1 | 0.3000 | 1.0740 | 0.0210 | 0.0215 | 24 | 0.5040 | 0.5155 | 1.0195 | 1.68 |
| Sodium Citrate tube (Coagulation) | Product | Polypropylene | 0.0080 | 0.20 | 1 | 1 | 0.3000 | 1.0740 | 0.0600 | 0.0086 | 50 | 3.0000 | 0.4296 | 3.4296 | 10.00 |
| Glucose POCT | Product | Polypropylene | 0.0120 | 0.24 | 1 | 1 | 0.3000 | 1.0720 | 0.0720 | 0.0129 | 160 | 11.5200 | 2.0582 | 13.5782 | 38.40 |
| Blood culture | Product | Polypropylene | 0.0360 | 4.60 | 1 | 1 | 0.3000 | 1.0740 | 1.3800 | 0.0387 | 11 | 15.1800 | 0.4253 | 15.6053 | 50.60 |
| Arterial blood gas | Product | Polypropylene | 0.0057 | 0.74 | 1 | 1 | 0.3000 | 1.0740 | 0.2220 | 0.0061 | 34 | 7.5480 | 0.2081 | 7.7561 | 25.16 |
|  | Packaging | LDPE | 0.0006 |  | 1 | 1 | 2.6000 | 0.1720 | 0.0016 | 0.0001 | 34 | 0.0530 | 0.0035 | 0.0565 | 0.00 |
| Blunt Fill Needle with filter | Product | stainless steel | 0.0010 | 0.13 | 1 | 1 | 0.3000 | 1.0740 | 0.0390 | 0.0011 | 2 | 0.0780 | 0.0021 | 0.0801 | 0.26 |
|  | Packaging | Paper | 0.0003 |  | 1 | 1 | 0.9190 | 0.1720 | 0.0003 | 0.0001 | 2 | 0.0006 | 0.0001 | 0.0007 | 0.00 |
|  | Packaging | LDPE | 0.0003 |  | 1 | 1 | 2.6006 | 0.1720 | 0.0008 | 0.0001 | 2 | 0.0016 | 0.0001 | 0.0017 | 0.00 |

| Pharmaceuticals | | | | | | | | | | | | | | | |
| --- | --- | --- | --- | --- | --- | --- | --- | --- | --- | --- | --- | --- | --- | --- | --- |
| Product | Component | Material | Weight (kg) | Cost (£) | Number of uses | Number of scenarios per use | Emissions factor | Waste stream Emission Factor | Production | Waste | Number | Production total | Waste total | Total emissions | Total Cost (£) |
| Flucloxacillin 500mg PO | Product | Pharmaceuticals |  | 0.11 | 1 | 1 | 0.1550 |  | 0.0167 |  | 672 | 11.1972 |  | 11.1972 | 72.24 |
|  | Packaging | Aluminium | 0.0002 |  | 1 | 1 | 9.1220 | 0.1720 | 0.0015 | 0.0000 | 672 | 1.0053 | 0.0190 | 1.0243 | 0.00 |
| Flucloxacillin 1g PO | Product | Pharmaceuticals |  | 0.22 | 1 | 1 | 0.1550 |  | 0.0333 |  | 55 | 1.8329 |  | 1.8329 | 11.83 |
|  | Packaging | Aluminium | 0.0002 |  | 1 | 1 | 9.1220 | 0.1720 | 0.0015 | 0.0000 | 55 | 0.0823 | 0.0016 | 0.0838 | 0.00 |
| Flucloxacillin 1g IV | Product | Pharmaceuticals |  | 3.45 | 1 | 1 | 0.1550 |  | 0.5348 |  | 29 | 15.5078 |  | 15.5078 | 100.05 |
|  | Packaging | LDPE | 0.0240 |  | 1 | 1 | 2.6006 | 0.1720 | 0.0624 | 0.0041 | 29 | 1.8100 | 0.1197 | 1.9298 | 0.00 |
| Flucloxacillin 2g IV | Product | Pharmaceuticals |  | 6.00 | 1 | 1 | 0.1550 |  | 0.9300 |  | 40 | 37.2000 |  | 37.2000 | 240.00 |
|  | Packaging | LDPE | 0.0240 |  | 1 | 1 | 2.6006 | 0.1720 | 0.0624 | 0.0041 | 40 | 2.4966 | 0.1651 | 2.6617 | 0.00 |
| Metronidazole 400mg PO | Product | Pharmaceuticals |  | 0.06 | 1 | 1 | 0.1550 |  | 0.0098 | 0.0000 | 231 | 2.2677 | 0.0000 | 2.2677 | 14.63 |
|  | Packaging | Aluminium | 0.0002 |  | 1 | 1 | 9.1220 | 0.1720 | 0.0021 | 0.0000 | 231 | 0.4937 | 0.0093 | 0.5030 | 0.00 |
| Metronidazole 500mg IV | Product | Pharmaceuticals |  | 3.59 | 1 | 1 | 0.1550 |  | 0.5568 |  | 8 | 4.4547 |  | 4.4547 | 28.74 |
|  | Packaging | LDPE | 0.0240 |  | 1 | 1 | 2.6006 | 0.1720 | 0.0624 | 0.0041 | 8 | 0.4993 | 0.0330 | 0.5323 | 0.00 |
| Doxycycline 100mg | Product | Pharmaceuticals |  | 0.11 | 1 | 1 | 0.1550 |  | 0.0167 |  | 28 | 0.4666 |  | 0.4666 | 3.01 |
|  | Packaging | Aluminium | 0.0002 |  | 1 | 1 | 9.1220 | 0.1720 | 0.0015 | 0.0000 | 28 | 0.0419 | 0.0008 | 0.0427 | 0.00 |
| Co-trimoxazole 960mg PO | Product | Pharmaceuticals |  | 0.24 | 1 | 1 | 0.1550 |  | 0.0367 | 0.0000 | 308 | 11.3144 | 0.0000 | 11.3144 | 73.00 |
|  | Packaging | Aluminium | 0.0002 |  | 1 | 1 | 9.1220 | 0.1720 | 0.0015 | 0.0000 | 308 | 0.4608 | 0.0087 | 0.4695 | 0.00 |
| Clarithromycin 500mg IV | Product | Pharmaceuticals |  | 11.15 | 1 | 1 | 0.1550 |  | 1.7283 |  | 42 | 72.5865 | 0.0000 | 72.5865 | 468.30 |
|  | Packaging | LDPE | 0.0240 |  | 1 | 1 | 2.6006 | 0.1720 | 0.0624 | 0.0041 | 42 | 2.6214 | 0.1734 | 2.7948 | 0.00 |
| Gentamicin 240mg IV | Product | Pharmaceuticals |  | 3.60 | 1 | 1 | 0.1550 |  | 0.5580 |  | 1 | 0.5580 |  | 0.5580 | 3.60 |
|  | Packaging | LDPE | 0.0240 |  | 1 | 1 | 2.6006 | 0.1720 | 0.0624 | 0.0041 | 1 | 0.0624 | 0.0041 | 0.0665 | 0.00 |
| Gentamicin 320mg IV | Product | Pharmaceuticals |  | 7.22 | 1 | 1 | 0.1550 |  | 1.1198 |  | 2 | 2.2396 |  | 2.2396 | 14.45 |
|  | Packaging | LDPE | 0.0240 |  | 1 | 1 | 2.6006 | 0.1720 | 0.0624 | 0.0041 | 2 | 0.1248 | 0.0083 | 0.1331 | 0.00 |
| Teicoplanin 400mg IV | Product | Pharmaceuticals |  | 7.32 | 1 | 1 | 0.1550 |  | 1.1346 |  | 4 | 4.5384 |  | 4.5384 | 29.28 |
|  | Packaging | LDPE | 0.0240 |  | 1 | 1 | 2.6006 | 0.1720 | 0.0624 | 0.0041 | 4 | 0.2497 | 0.0165 | 0.2662 | 0.00 |
| Teicoplanin 800mg IV | Product | Pharmaceuticals |  | 18.16 | 1 | 1 | 0.1550 |  | 2.8148 |  | 18 | 50.6664 |  | 50.6664 | 326.88 |
|  | Packaging | LDPE | 0.0240 |  | 1 | 1 | 2.6006 | 0.1720 | 0.0624 | 0.0041 | 18 | 1.1235 | 0.0743 | 1.1978 | 0.00 |
| Teicoplanin 2g IV | Product | Pharmaceuticals |  | 36.60 | 1 | 1 | 0.1550 |  | 5.6730 |  | 3 | 17.0190 |  | 17.0190 | 109.80 |
|  | Packaging | LDPE | 0.0240 |  | 1 | 1 | 2.6006 | 0.1720 | 0.0624 | 0.0041 | 3 | 0.1872 | 0.0124 | 0.1996 | 0.00 |
| Cefuroxime 750mg IV | Product | Pharmaceuticals |  | 2.52 | 1 | 1 | 0.1550 |  | 0.3906 |  | 6 | 2.3436 |  | 2.3436 | 15.12 |
|  | Packaging | LDPE | 0.0240 |  | 1 | 1 | 2.6006 | 0.1720 | 0.0624 | 0.0041 | 6 | 0.3745 | 0.0248 | 0.3993 | 0.00 |
| Piperacillin and Tazobactam 4.5g IV | Product | Pharmaceuticals |  | 15.75 | 1 | 1 | 0.1550 |  | 2.4413 |  | 9 | 21.9713 |  | 21.9713 | 141.75 |
|  | Packaging | LDPE | 0.0240 |  | 1 | 1 | 2.6006 | 0.1720 | 0.0624 | 0.0041 | 9 | 0.5617 | 0.0372 | 0.5989 | 0.00 |
| Linezolid 600mg PO | Product | Pharmaceuticals |  | 32.72 | 1 | 1 | 0.1550 |  | 5.0711 |  | 49 | 248.4856 |  | 248.4856 | 1603.13 |
|  | Packaging | Aluminium | 0.0002 |  | 1 | 1 | 9.1220 | 0.1720 | 0.0015 | 0.0000 | 49 | 0.0733 | 0.0014 | 0.0747 | 0.00 |
| Linezolid 600mg IV | Product | Pharmaceuticals |  | 44.50 | 1 | 1 | 0.1550 |  | 6.8975 |  | 5 | 34.4875 |  | 34.4875 | 222.50 |
|  | Packaging | LDPE | 0.0240 |  | 1 | 1 | 2.6006 | 0.1720 | 0.0624 | 0.0041 | 5 | 0.3121 | 0.0206 | 0.3327 | 0.00 |
| Meropenem 1g IV | Product | Pharmaceuticals |  | 16.00 | 1 | 1 | 0.1550 |  | 2.4800 |  | 13 | 32.2400 |  | 32.2400 | 208.00 |
|  | Packaging | LDPE | 0.0240 |  | 1 | 1 | 2.6006 | 0.1720 | 0.0624 | 0.0041 | 13 | 0.8114 | 0.0537 | 0.8651 | 0.00 |
| Meropenem 2g IV | Product | Pharmaceuticals |  | 32.00 | 1 | 1 | 0.1550 |  | 4.9600 |  | 96 | 476.1600 |  | 476.1600 | 3072.00 |
|  | Packaging | LDPE | 0.0240 |  | 1 | 1 | 2.6006 | 0.1720 | 0.0624 | 0.0041 | 96 | 5.9919 | 0.3963 | 6.3882 | 0.00 |
| Ertapenem1g IV | Product | Pharmaceuticals |  | 31.65 | 1 | 1 | 0.1550 |  | 4.9058 |  | 49 | 240.3818 |  | 240.3818 | 1550.85 |
|  | Packaging | LDPE | 0.0240 |  | 1 | 1 | 2.6006 | 0.1720 | 0.0624 | 0.0041 | 49 | 3.0584 | 0.2023 | 3.2606 | 0.00 |
| Ciprofloxacin 750mg PO | Product | Pharmaceuticals |  | 1.05 | 1 | 1 | 0.1550 |  | 0.1629 |  | 56 | 9.1227 |  | 9.1227 | 58.86 |
|  | Packaging | Aluminium | 0.0002 |  | 1 | 1 | 9.1220 | 0.1720 | 0.0015 | 0.0000 | 56 | 0.0838 | 0.0016 | 0.0854 | 0.00 |
| Co-amoxiclav 625mg PO | Product | Pharmaceuticals |  | 0.29 | 1 | 1 | 0.1550 |  | 0.0443 |  | 63 | 2.7900 |  | 2.7900 | 18.00 |
|  | Packaging | Aluminium | 0.0002 |  | 1 | 1 | 9.1220 | 0.1720 | 0.0021 | 0.0000 | 63 | 0.1346 | 0.0025 | 0.1372 | 0.00 |
| Co-amoxiclav 1.2g IV | Product | Pharmaceuticals |  | 2.75 | 1 | 1 | 0.1550 |  | 0.4263 |  | 4 | 1.7050 |  | 1.7050 | 11.00 |
|  | Packaging | LDPE | 0.0240 |  | 1 | 1 | 2.6006 | 0.1720 | 0.0624 | 0.0041 | 4 | 0.2497 | 0.0165 | 0.2662 | 0.00 |
| Aztreonam 2g IV | Product | Pharmaceuticals |  | 18.82 | 1 | 1 | 0.1550 |  | 2.9171 |  | 3 | 8.7513 |  | 8.7513 | 56.46 |
|  | Packaging | LDPE | 0.0240 |  | 1 | 1 | 2.6006 | 0.1720 | 0.0624 | 0.0041 | 3 | 0.1872 | 0.0124 | 0.1996 | 0.00 |
| Moxifloxacin 400mg PO | Product | Pharmaceuticals |  | 2.49 | 1 | 1 | 0.1550 |  | 0.3853 |  | 13 | 5.0093 |  | 5.0093 | 32.32 |
|  | Packaging | Aluminium | 0.0002 |  | 1 | 1 | 9.1220 | 0.1720 | 0.0015 | 0.0000 | 13 | 0.0194 | 0.0004 | 0.0198 | 0.00 |
| Paracetamol 1g PO | Product | Pharmaceuticals |  | 0.05 | 1 | 1 | 0.1550 |  | 0.0079 |  | 353 | 2.8014 |  | 2.8014 | 18.07 |
|  | Packaging | Aluminium | 0.0002 |  | 1 | 1 | 9.1220 | 0.1720 | 0.0015 | 0.0000 | 353 | 0.5281 | 0.0100 | 0.5380 | 0.00 |
| Paracetamol 1g IV | Product | Pharmaceuticals |  | 1.35 | 1 | 1 | 0.1550 |  | 0.2093 |  | 26 | 5.4405 |  | 5.4405 | 35.10 |
|  | Packaging | LDPE | 0.0240 |  | 1 | 1 | 2.6006 | 0.1720 | 0.0624 | 0.0041 | 26 | 1.6228 | 0.1073 | 1.7301 | 0.00 |
| Co-codamol 30.500mg | Product | Pharmaceuticals |  | 0.04 | 1 | 1 | 0.1550 |  | 0.0063 |  | 95 | 0.5993 |  | 0.5993 | 3.87 |
|  | Packaging | Aluminium | 0.0002 |  | 1 | 1 | 9.1220 | 0.1720 | 0.0015 | 0.0000 | 95 | 0.1421 | 0.0027 | 0.1448 | 0.00 |
| Codeine Phosphate 30mg PO | Product | Pharmaceuticals |  | 0.04 | 1 | 1 | 0.1550 |  | 0.0055 | 0.0000 | 120 | 0.6576 | 0.0000 | 0.6576 | 4.24 |
|  | Packaging | Aluminium | 0.0002 |  | 1 | 1 | 9.1220 | 0.1720 | 0.0015 | 0.0000 | 120 | 0.1795 | 0.0034 | 0.1829 | 0.00 |
| Codeine Phosphate 60mg PO | Product | Pharmaceuticals |  | 0.07 | 1 | 1 | 0.1550 |  | 0.0106 | 0.0000 | 1 | 0.0106 | 0.0000 | 0.0106 | 0.07 |
|  | Packaging | Aluminium | 0.0002 |  | 1 | 1 | 9.1220 | 0.1720 | 0.0015 | 0.0000 | 1 | 0.0015 | 0.0000 | 0.0015 | 0.00 |
| Dihydrocodeine | Product | Pharmaceuticals |  | 0.09 | 1 | 1 | 0.1550 |  | 0.0141 |  | 3 | 0.0423 |  | 0.0423 | 0.27 |
|  | Packaging | Aluminium | 0.0002 |  | 1 | 1 | 9.1220 | 0.1720 | 0.0015 | 0.0000 | 3 | 0.0045 | 0.0001 | 0.0046 | 0.00 |
| Oxycodone 10mg MR | Product | Pharmaceuticals |  | 0.17 | 1 | 1 | 0.1550 |  | 0.0260 | 0.0000 | 6 | 0.1559 | 0.0000 | 0.1559 | 1.01 |
|  | Packaging | Aluminium | 0.0002 |  | 1 | 1 | 9.1220 | 0.1720 | 0.0015 | 0.0000 | 6 | 0.0090 | 0.0002 | 0.0091 | 0.00 |
| Oxycodone 10mg IR | Product | Pharmaceuticals |  | 0.25 | 1 | 1 | 0.1550 |  | 0.0380 | 0.0000 | 1 | 0.0380 | 0.0000 | 0.0380 | 0.25 |
|  | Packaging | Aluminium | 0.0002 |  | 1 | 1 | 9.1220 | 0.1720 | 0.0015 | 0.0000 | 1 | 0.0015 | 0.0000 | 0.0015 | 0.00 |
| MST 5mg | Product | Pharmaceuticals |  | 0.05 | 1 | 1 | 0.1550 |  | 0.0085 |  | 10 | 0.0850 |  | 0.0850 | 0.55 |
|  | Packaging | Aluminium | 0.0002 |  | 1 | 1 | 9.1220 | 0.1720 | 0.0015 | 0.0000 | 10 | 0.0150 | 0.0003 | 0.0152 | 0.00 |
| MST 10mg | Product | Pharmaceuticals |  | 0.09 | 1 | 1 | 0.1550 |  | 0.0134 |  | 10 | 0.1343 |  | 0.1343 | 0.87 |
|  | Packaging | Aluminium | 0.0002 |  | 1 | 1 | 9.1220 | 0.1720 | 0.0015 | 0.0000 | 10 | 0.0150 | 0.0003 | 0.0152 | 0.00 |
| MST 15mg | Product | Pharmaceuticals |  | 0.15 | 1 | 1 | 0.1550 |  | 0.0235 |  | 61 | 1.4340 |  | 1.4340 | 9.25 |
|  | Packaging | Aluminium | 0.0002 |  | 1 | 1 | 9.1220 | 0.1720 | 0.0015 | 0.0000 | 61 | 0.0913 | 0.0017 | 0.0930 | 0.00 |
| Morphine 10mg/5ml Oral Solution | Product | Pharmaceuticals |  | 0.08 | 1 | 1 | 0.1550 |  | 0.0116 | 0.0000 | 653 | 7.5940 | 0.0000 | 7.5940 | 48.99 |
|  | Packaging | Glass | 0.0048 |  | 1 | 1 | 1.4020 | 1.0740 | 0.0067 | 0.0052 | 504 | 3.3917 | 2.5982 | 5.9899 | 0.00 |
| Morhpine 1mg/ml PCA infusion | Product | Pharmaceuticals |  | 0.18 | 1 | 1 | 0.1550 |  | 0.0271 | 0.0000 | 399 | 10.8283 | 0.0000 | 10.8283 | 69.86 |
|  | Packaging | Glass | 0.0005 |  | 1 | 1 | 1.4020 | 1.0740 | 0.0007 | 0.0005 | 399 | 0.2686 | 0.2058 | 0.4744 | 0.00 |
| Morphine 10mg/10ml x1ml | Product | Pharmaceuticals |  | 1.75 | 1 | 1 | 0.1550 |  | 0.2713 | 0.0000 | 6 | 1.4919 | 0.0000 | 1.4919 | 9.63 |
|  | Packaging | Glass | 0.0048 |  | 1 | 1 | 1.4020 | 1.0740 | 0.0067 | 0.0052 | 6 | 0.0370 | 0.0284 | 0.0654 | 0.00 |
| Levobupivicaine 0.25% 25mg/20ml (1ml) | Product | Pharmaceuticals |  | 0.21 | 1 | 1 | 0.1550 |  | 0.0328 | 0.0000 | 3 | 0.0820 | 0.0000 | 0.0820 | 0.53 |
|  | Packaging | LDPE | 0.0002 |  | 1 | 1 | 1.4020 | 1.0740 | 0.0003 | 0.0003 | 3 | 0.0008 | 0.0006 | 0.0015 | 0.00 |
| Nefopam 30mg PO | Product | Pharmaceuticals |  | 0.04 | 1 | 1 | 0.1550 |  | 0.0068 | 0.0000 | 122 | 0.8341 | 0.0000 | 0.8341 | 5.38 |
|  | Packaging | Aluminium | 0.0002 |  | 1 | 1 | 9.1220 | 0.1720 | 0.0015 | 0.0000 | 122 | 0.1825 | 0.0034 | 0.1860 | 0.00 |
| Dalteparin 18,000 units | Product | Pharmaceuticals |  | 10.16 | 1 | 1 | 0.1550 |  | 1.5754 |  | 22 | 34.6592 |  | 34.6592 | 223.61 |
|  | Packaging | LDPE | 0.0240 |  | 1 | 1 | 2.6006 | 0.1720 | 0.0624 | 0.0041 | 22 | 1.3731 | 0.0908 | 1.4640 | 0.00 |
| Dalteparin 12500 units | Product | Pharmaceuticals |  | 7.06 | 1 | 1 | 0.1550 |  | 1.0940 |  | 2 | 2.1880 |  | 2.1880 | 14.12 |
|  | Packaging | LDPE | 0.0240 |  | 1 | 1 | 2.6006 | 0.1720 | 0.0624 | 0.0041 | 2 | 0.1248 | 0.0083 | 0.1331 | 0.00 |
| Dalteparin 5000 units | Product | Pharmaceuticals |  | 2.82 | 1 | 1 | 0.1550 |  | 0.4376 |  | 167 | 73.0734 |  | 73.0734 | 471.44 |
|  | Packaging | LDPE | 0.0240 |  | 1 | 1 | 2.6006 | 0.1720 | 0.0624 | 0.0041 | 167 | 10.4234 | 0.6894 | 11.1127 | 0.00 |
| Pregabalin 300mg | Product | Pharmaceuticals |  | 0.13 | 1 | 1 | 0.1550 |  | 0.0199 | 0.0000 | 22 | 0.4378 | 0.0000 | 0.4378 | 2.82 |
|  | Packaging | Aluminium | 0.0002 |  | 1 | 1 | 9.1220 | 0.1720 | 0.0015 | 0.0000 | 22 | 0.0329 | 0.0006 | 0.0335 | 0.00 |
| Pregabalin 225mg | Product | Pharmaceuticals |  | 0.11 | 1 | 1 | 0.1550 |  | 0.0177 | 0.0000 | 1 | 0.0177 | 0.0000 | 0.0177 | 0.11 |
|  | Packaging | Aluminium | 0.0002 |  | 1 | 1 | 9.1220 | 0.1720 | 0.0015 | 0.0000 | 1 | 0.0015 | 0.0000 | 0.0015 | 0.00 |
| Pregabalin 100mg | Product | Pharmaceuticals |  | 0.07 | 1 | 1 | 0.1550 |  | 0.0103 | 0.0000 | 6 | 0.0619 | 0.0000 | 0.0619 | 0.40 |
|  | Packaging | Aluminium | 0.0002 |  | 1 | 1 | 9.1220 | 0.1720 | 0.0015 | 0.0000 | 6 | 0.0090 | 0.0002 | 0.0091 | 0.00 |
| Pregabalin 50mg | Product | Pharmaceuticals |  | 0.05 | 1 | 1 | 0.1550 |  | 0.0074 | 0.0000 | 1 | 0.0074 | 0.0000 | 0.0074 | 0.05 |
|  | Packaging | Aluminium | 0.0002 |  | 1 | 1 | 9.1220 | 0.1720 | 0.0015 | 0.0000 | 1 | 0.0015 | 0.0000 | 0.0015 | 0.00 |
| Gabapentin 300mg PO | Product | Pharmaceuticals |  | 0.03 | 1 | 1 | 0.1550 |  | 0.0042 |  | 101 | 0.4289 |  | 0.4289 | 2.77 |
|  | Packaging | Aluminium | 0.0002 |  | 1 | 1 | 9.1220 | 0.1720 | 0.0021 | 0.0000 | 101 | 0.2159 | 0.0041 | 0.2199 | 0.00 |
| Iloprost 50mcg | Product | Pharmaceuticals |  | 75.00 | 1 | 1 | 0.1550 |  | 11.6250 | 0.0000 | 1 | 11.6250 | 0.0000 | 11.6250 | 75.00 |
|  | Packaging | LDPE | 0.0002 |  | 1 | 1 | 1.4020 | 1.0740 | 0.0003 | 0.0003 | 1 | 0.0003 | 0.0003 | 0.0006 | 0.00 |
| Ondansetron 4mg IV | Product | Pharmaceuticals | 0.0002 | 5.99 | 1 | 1 | 0.1550 |  | 0.9285 | 0.0000 | 18 | 16.7121 | 0.0000 | 16.7121 | 107.82 |
|  | Packaging | Glass | 0.0048 |  | 1 | 1 | 1.4020 | 1.0740 | 0.0067 | 0.0052 | 18 | 0.1211 | 0.0928 | 0.2139 | 0.00 |
| Ondansetron 8mg IV | Product | Pharmaceuticals | 0.0002 | 5.67 | 1 | 1 | 0.1550 |  | 0.8795 | 0.0000 | 6 | 5.2768 | 0.0000 | 5.2768 | 34.04 |
|  | Packaging | Glass | 0.0048 |  | 1 | 1 | 1.4020 | 1.0740 | 0.0067 | 0.0052 | 6 | 0.0404 | 0.0309 | 0.0713 | 0.00 |
| Cyclizine 50mg IV | Product | Pharmaceuticals |  | 1.95 | 1 | 1 | 0.1550 |  | 0.3019 |  | 25 | 7.5485 |  | 7.5485 | 48.70 |
|  | Packaging | LDPE | 0.0240 |  | 1 | 1 | 2.6006 | 0.1720 | 0.0624 | 0.0041 | 25 | 1.5604 | 0.1032 | 1.6636 | 0.00 |
| Metoclopramide 10mg IV | Product | Pharmaceuticals |  | 0.90 | 1 | 1 | 0.1550 |  | 0.1395 |  | 1 | 0.1395 |  | 0.1395 | 0.90 |
|  | Packaging | LDPE | 0.0240 |  | 1 | 1 | 2.6006 | 0.1720 | 0.0624 | 0.0041 | 1 | 0.0624 | 0.0041 | 0.0665 | 0.00 |
| Harmanns 1L | Product | Pharmaceuticals |  | 2.21 | 1 | 1 | 0.1550 |  | 0.4108 |  | 4 | 0.4108 | 0.0000 | 0.4108 | 7.74 |
|  | Packaging | LDPE | 0.0500 |  | 1 | 1 | 2.6010 | 0.1720 | 0.1301 | 0.0086 | 4 | 0.1301 | 0.0011 | 0.1312 | 0.00 |
|  | Packaging | LDPE | 0.0102 |  | 1 | 1 | 2.6000 | 0.1720 | 0.0265 | 0.0018 | 4 | 0.0928 | 0.0061 | 0.0990 | 0.00 |
| NaCl 1L | Product | Pharmaceuticals |  | 2.65 | 1 | 1 | 0.1550 |  | 0.4108 |  | 3 | 1.2323 | 0.0000 | 1.2323 | 7.95 |
|  | Packaging | LDPE | 0.0500 |  | 1 | 1 | 2.6010 | 0.1720 | 0.1301 | 0.0086 | 3 | 0.3902 | 0.0034 | 0.3935 | 0.00 |
|  | Packaging | LDPE | 0.0102 |  | 1 | 1 | 2.6000 | 0.1720 | 0.0265 | 0.0018 | 3 | 0.0796 | 0.0053 | 0.0848 | 0.00 |
| 0.9% NaCL 500ml | Product | Pharmaceuticals | 0.5278 | 1.71 | 1 | 1 | 0.3000 |  | 0.5130 |  | 31 | 15.9030 | 0.0000 | 15.9030 | 53.01 |
|  | Packaging | LDPE | 0.0200 |  | 1 | 1 | 2.6000 | 0.1720 | 0.0520 | 0.0034 | 31 | 1.6120 | 0.1066 | 1.7186 | 0.00 |
|  | Packaging | LDPE | 0.0102 |  | 1 | 1 | 2.6000 | 0.1720 | 0.0265 | 0.0018 | 31 | 0.8221 | 0.0544 | 0.8765 | 0.00 |
| NaCl 250ml | Product | Pharmaceuticals |  | 1.85 | 1 | 1 | 0.1550 |  | 0.2868 |  | 1 | 0.2868 | 0.0000 | 0.2868 | 1.85 |
|  | Packaging | LDPE | 0.0500 |  | 1 | 1 | 2.6010 | 0.1720 | 0.1301 | 0.0086 | 1 | 0.1301 | 0.0011 | 0.1312 | 0.00 |
|  | Packaging | LDPE | 0.0102 |  | 1 | 1 | 2.6000 | 0.1720 | 0.0265 | 0.0018 | 1 | 0.0265 | 0.0018 | 0.0283 | 0.00 |
| Glucose 5% infusion 500ml | Product | Pharmaceuticals | 0.5278 | 1.88 | 1 | 1 | 0.3000 |  | 0.5640 |  | 1 | 0.5640 | 0.0000 | 0.5640 | 1.88 |
|  | Packaging | LDPE | 0.0200 |  | 1 | 1 | 2.6000 | 0.1720 | 0.0520 | 0.0034 | 1 | 0.0520 | 0.0034 | 0.0554 | 0.00 |
|  | Packaging | LDPE | 0.0102 |  | 1 | 1 | 2.6000 | 0.1720 | 0.0265 | 0.0018 | 1 | 0.0265 | 0.0018 | 0.0283 | 0.00 |
| Senna 15mg PO | Product | Pharmaceuticals |  | 0.08 | 1 | 1 | 0.1550 |  | 0.0124 | 0.0000 | 5 | 0.0620 | 0.0000 | 0.0620 | 0.40 |
|  | Packaging | Aluminium | 0.0002 |  | 1 | 1 | 9.1220 | 0.1720 | 0.0015 | 0.0000 | 5 | 0.0075 | 0.0001 | 0.0076 | 0.00 |
| Phosphates enema 128ml | Product | Pharmaceuticals | 0.1280 | 30.78 | 1 | 1 | 0.1550 |  | 4.7709 | 0.0000 | 1 | 4.7709 | 0.0000 | 4.7709 | 30.78 |
|  | Packaging | Aluminium | 0.0460 |  | 1 | 1 | 9.1220 | 0.1720 | 0.4196 | 0.0079 | 1 | 0.4196 | 0.0079 | 0.4275 | 0.00 |
| Lactulose 15ml | Product | Pharmaceuticals |  | 0.25 | 1 | 1 | 0.1550 |  | 0.0388 | 0.0000 | 37 | 1.4338 | 0.0000 | 1.4338 | 9.25 |
|  | Packaging | Paper | 0.0069 |  | 1 | 1 | 0.9190 | 0.1720 | 0.0063 | 0.0012 | 37 | 0.2347 | 0.0439 | 0.2786 | 0.00 |
| Macrogol | Product | Pharmaceuticals |  | 4.64 | 1 | 1 | 0.1550 |  | 0.7186 |  | 51 | 36.6481 |  | 36.6481 | 236.44 |
|  | Packaging | Paper | 0.0089 |  | 1 | 1 | 9.1220 | 0.1720 | 0.0814 | 0.0015 | 51 | 4.1526 | 0.0783 | 4.2309 | 0.00 |
| Zopiclone 7.5mg PO | Product | Pharmaceuticals |  | 0.08 | 1 | 1 | 0.1550 |  | 0.0125 | 0.0000 | 12 | 0.1495 | 0.0000 | 0.1495 | 0.96 |
|  | Packaging | Aluminium | 0.0002 |  | 1 | 1 | 9.1220 | 0.1720 | 0.0015 | 0.0000 | 12 | 0.0180 | 0.0003 | 0.0183 | 0.00 |
| Nicotine 21mg Transderm patch | Product | Pharmaceuticals |  | 1.64 | 1 | 1 | 0.1550 |  | 0.2542 | 0.0000 | 4 | 1.0168 | 0.0000 | 1.0168 | 6.56 |
|  | Packaging | Aluminium | 0.0002 |  | 1 | 1 | 9.1220 | 0.1720 | 0.0015 | 0.0000 | 4 | 0.0060 | 0.0001 | 0.0061 | 0.00 |
| Bumetanide 1mg | Product | Pharmaceuticals |  | 0.06 | 1 | 1 | 0.1550 |  | 0.0086 |  | 7 | 0.0601 |  | 0.0601 | 0.39 |
|  | Packaging | Aluminium | 0.0002 |  | 1 | 1 | 9.1220 | 0.1720 | 0.0021 | 0.0000 | 7 | 0.0150 | 0.0003 | 0.0152 | 0.00 |
| Insulin aspart biphasic - novomix 30 flexpen 40 units | Product | Pharmaceuticals |  | 0.82 | 1 | 1 | 0.1550 |  | 0.1265 |  | 23 | 2.9090 |  | 2.9090 | 18.77 |
|  | Packaging | LDPE | 0.0240 |  | 1 | 1 | 2.6006 | 0.1720 | 0.0624 | 0.0041 | 23 | 1.4356 | 0.0949 | 1.5305 | 0.00 |
| Lidocaine Hydrochloride 10mg/ml (1%) 1ml | Product | Pharmaceuticals | 0.0063 | 0.11 | 1 | 1 | 0.3000 |  | 0.0336 |  | 3 | 0.1008 | 0.0000 | 0.1008 | 0.34 |
|  | Packaging | Polypropylene | 0.0013 |  | 1 | 1 | 3.1050 | 0.1720 | 0.0040 | 0.0002 | 3 | 0.0121 | 0.0007 | 0.0128 | 0.00 |
| Alteplase 2mg (10mg vial) | Product | Pharmaceuticals |  | 172.80 | 1 | 1 | 0.1550 |  | 26.7840 | 0.0000 | 3 | 80.3520 | 0.0000 | 80.3520 | 518.40 |
|  | Packaging | Glass | 0.0048 |  | 1 | 1 | 1.4020 | 1.0740 | 0.0067 | 0.0052 | 3 | 0.0202 | 0.0155 | 0.0357 | 0.00 |

| Procedures | | | | | | | | | | | | | | | |
| --- | --- | --- | --- | --- | --- | --- | --- | --- | --- | --- | --- | --- | --- | --- | --- |
| Product | Component | Material | Weight (kg) | Cost (£) | Number of uses | Number of scenarios per use | Emissions factor | Waste stream Emission Factor | Production | Waste | Number | Production total | Waste total | Total emissions | Cost |
| Consultant anaesthetist | Review |  |  | 113.00 | 1 |  |  |  |  |  | 26 |  |  |  | 2966.25 |
| Consultant Radiologist | Review |  |  | 113.00 | 1 |  |  |  |  |  | 4 |  |  |  | 452.00 |
| Registrar | Review |  |  | 53.00 | 1 |  |  |  |  |  | 28 |  |  |  | 1497.25 |
| Consultant surgeon | Review |  |  | 111.00 | 1 |  |  |  |  |  | 26 |  |  |  | 2913.75 |
| Band 5 staff | Review |  |  | 43.00 | 3 |  |  |  |  |  | 36 |  |  |  | 1558.75 |
| 5F Nylex Sheath Terumo | Product | Nylon/polyamide | 0.0480 | 12.96 | 1 | 1 | 0.4100 | 1.0740 | 5.3136 | 0.0516 | 3 | 15.9408 | 0.1547 | 16.0955 | 38.88 |
|  | Packaging | Paper | 0.0010 |  | 1 | 1 | 0.9200 | 0.1720 | 0.0009 | 0.0002 | 3 | 0.0028 | 0.0005 | 0.0033 | 0.00 |
|  | Packaging | LDPE | 0.0010 |  | 1 | 1 | 2.6000 | 0.1720 | 0.0026 | 0.0002 | 3 | 0.0078 | 0.0005 | 0.0083 | 0.00 |
| Omnipaque 300 mg/ml (100ml) (total weight) | Product | Iohexol | 0.1680 |  | 1 | 1 | 0.3000 |  | 0.0000 | 0.0000 | 3 | 0.0000 | 0.0000 | 0.0000 | 0.00 |
|  | Packaging | Polypropylene | 0.0240 |  | 1 | 1 | 0.3000 | 0.1720 | 0.0072 | 0.0041 | 3 | 0.0216 | 0.0124 | 0.0340 | 0.00 |
| 6F Angioseal Terumo | Product | collagen | 0.0040 | 166.80 | 1 | 1 | 0.4100 |  | 68.3880 |  | 3 | 205.1640 | 0.0000 | 205.1640 | 500.40 |
|  | Product | Nylon/polyamide | 0.0700 |  | 1 | 1 |  | 1.0740 |  | 0.0752 | 3 | 0.0000 | 0.2255 | 0.2255 | 0.00 |
|  | Packaging | LDPE | 0.0220 |  | 1 | 1 | 2.6000 | 0.1720 | 0.0572 | 0.0038 | 3 | 0.1716 | 0.0114 | 0.1830 | 0.00 |
| Scalpel 15 | Product | Steel | 1.0010 | 0.27 | 1 | 1 | 0.3000 | 1.0740 | 0.0810 | 0.0448 | 3 | 0.2430 | 0.1344 | 0.3774 | 0.81 |
| Needle 21G green | Product | Polypropylene + steel |  | 0.08 | 1 | 1 | 0.3000 |  | 0.0240 | 0.0448 | 3 | 0.0720 | 0.1344 | 0.2064 | 0.24 |
| Towel paper | Product | Paper |  | 1.51 | 1 | 1 | 0.3000 |  | 0.0906 | 0.0448 | 3 | 0.2718 | 0.1344 | 0.4062 | 4.53 |
| Gallipot 120ml (x2) | Product | Polypropylene |  |  | 1 | 1 |  |  | 0.0906 | 0.0448 | 3 | 0.2718 | 0.1344 | 0.4062 | 0.00 |
| Sponge bowl 500ml (x2) | Product | Polypropylene |  |  | 1 | 1 |  |  | 0.0906 | 0.0448 | 3 | 0.2718 | 0.1344 | 0.4062 | 0.00 |
| Kidney bowl 700ml | Product | Polypropylene |  |  | 1 | 1 |  |  | 0.0906 | 0.0448 | 3 | 0.2718 | 0.1344 | 0.4062 | 0.00 |
| Bowl sponge | Product | rubber |  |  | 1 | 1 |  |  | 0.0906 | 0.0448 | 3 | 0.2718 | 0.1344 | 0.4062 | 0.00 |
| Needle containment device | Product | Polypropylene |  | 1.55 | 1 | 1 | 0.3000 |  | 0.4650 | 0.0448 | 3 | 1.3950 | 0.1344 | 1.5294 | 4.65 |
| 10ml Syringe (x3) | Product | Polypropylene |  | 0.30 | 1 | 1 | 0.3000 |  | 0.0900 | 0.0448 | 3 | 0.2700 | 0.1344 | 0.4044 | 0.90 |
| Foam prep spone 5cm x 4cm x 3cm | Product | Polypropylene + |  | 0.34 | 1 | 1 | 0.3000 |  | 0.1020 | 0.0448 | 3 | 0.3060 | 0.1344 | 0.4404 | 1.02 |
| Chloraprep tint 10.5ml | Product | Polypropylene |  | 1.10 | 1 | 1 | 0.3000 |  | 0.1100 | 0.0448 | 3 | 0.3300 | 0.1344 | 0.4644 | 3.30 |
|  | Product | Chlorhexadine gluconate 2% w/v and isopropyl alcohol 70% v/v |  |  | 1 | 1 |  |  | 0.1100 | 0.0448 | 3 | 0.3300 | 0.1344 | 0.4644 | 0.00 |
| PIL chloraprep tint | Product | Paper |  |  | 1 | 1 |  |  | 0.1100 | 0.0448 | 3 | 0.3300 | 0.1344 | 0.4644 | 0.00 |
| Guidewire bowl | Product | Polypropylene |  | 2.53 | 1 | 1 | 0.3000 |  | 0.7590 | 0.0448 | 3 | 2.2770 | 0.1344 | 2.4114 | 7.59 |
| Needle 25G orange | Product | Polypropylene + steel |  | 0.08 | 1 | 1 | 0.3000 |  | 0.0240 | 0.0448 | 3 | 0.0720 | 0.1344 | 0.2064 | 0.24 |
| Sponge stick medium 15cm | Product | Polypropylene + |  | 0.34 | 1 | 1 | 0.3000 |  | 0.1020 | 0.0448 | 3 | 0.3060 | 0.1344 | 0.4404 | 1.02 |
| Gauze swab 10cm x 10cm x4 (5 pack) | Product | Cotton |  | 0.55 | 1 | 1 | 0.3000 |  | 0.1650 | 0.0448 | 3 | 0.4950 | 0.1344 | 0.6294 | 1.65 |
| Trolley cover 140x140cm | Product | LDPE |  | 4.50 | 1 | 1 | 0.3000 |  | 1.3500 | 0.0448 | 3 | 4.0500 | 0.1344 | 4.1844 | 13.50 |
| 20ml syringe (x2) | Product | Polypropylene |  | 0.14 | 1 | 1 | 0.3000 |  | 0.0420 | 0.0448 | 3 | 0.1260 | 0.1344 | 0.2604 | 0.42 |
| 18G needle introducer 70mm | Product | Steel + LDPE + polypropylene |  | 1.80 | 1 | 1 | 0.3000 |  | 0.5400 | 0.0448 | 3 | 1.6200 | 0.1344 | 1.7544 | 5.40 |
| Cover fluoro medium blue | Product | Polypropylene |  | 1.54 | 1 | 1 | 0.3000 |  | 0.4620 | 0.0448 | 3 | 1.3860 | 0.1344 | 1.5204 | 4.62 |
| Drape 200x330cm | Product | LDPE |  | 10.70 | 1 | 1 | 0.3000 |  | 3.2100 | 0.0448 | 3 | 9.6300 | 0.1344 | 9.7644 | 32.10 |
| Clamp towl | Product | Cotton |  |  | 1 | 1 |  |  |  | 0.0448 | 3 | 0.0000 | 0.1344 | 0.1344 | 0.00 |
| Label waterproof 2 across 45x10mm (x40) | Product | Paper |  | 0.01 | 1 | 1 | 0.3000 |  | 0.0030 | 0.0448 | 3 | 0.0090 | 0.1344 | 0.1434 | 0.03 |
|  |  | Paper | 0.0130 |  | 1 | 1 | 0.9200 | 0.1720 | 0.0120 | 0.0022 | 3 | 0.0359 | 0.0067 | 0.0426 | 0.00 |
|  | Packaging | LDPE | 0.0460 |  | 1 | 1 | 2.6000 | 0.1720 | 0.1196 | 0.0079 | 3 | 0.3588 | 0.0237 | 0.3825 | 0.00 |
| Guidewire | Product | Nitinol (nickel titanium) | 0.0090 | 30.72 | 1 | 1 | 0.3000 | 1.0740 | 9.2160 | 0.0097 | 3 | 27.6480 | 0.0290 | 27.6770 | 92.16 |
|  | Packaging | Paper | 0.0050 |  | 1 | 1 | 0.9200 | 0.1720 | 0.0046 | 0.0009 | 3 | 0.0138 | 0.0026 | 0.0164 | 0.00 |
|  | Packaging | LDPE | 0.0040 |  | 1 | 1 | 2.6000 | 0.1720 | 0.0104 | 0.0007 | 3 | 0.0312 | 0.0021 | 0.0333 | 0.00 |
| Inflation device (basixCOMPAK) | Product | Polycarbonate | 0.1661 | 22.02 | 1 | 1 | 0.3000 | 1.0740 | 6.6060 | 0.1784 | 3 | 19.8180 | 0.5352 | 20.3532 | 66.06 |
|  | Packaging | Paper | 0.0039 |  | 1 | 1 | 0.9200 | 0.1720 | 0.0036 | 0.0007 | 3 | 0.0108 | 0.0020 | 0.0128 | 0.00 |
|  | Packaging | LDPE | 0.0500 |  | 1 | 1 | 2.6000 | 0.1720 | 0.1300 | 0.0086 | 3 | 0.3900 | 0.0258 | 0.4158 | 0.00 |
| Giving set (merit Disposal Depot) | Product | Polyurethane | 0.1298 |  | 1 | 1 | 0.3000 | 1.0740 | 0.0389 | 0.1394 | 3 | 0.1168 | 0.4182 | 0.5350 | 0.00 |
|  | Packaging | Paper | 0.0042 |  | 1 | 1 | 0.9200 | 0.1720 | 0.0039 | 0.0007 | 3 | 0.0116 | 0.0022 | 0.0138 | 0.00 |
|  | Packaging | LDPE | 0.0150 |  | 1 | 1 | 2.6000 | 0.1720 | 0.0390 | 0.0026 | 3 | 0.1170 | 0.0077 | 0.1247 | 0.00 |
| Torque device (terumo - radifocus) | Product | Polypropylene | 0.0010 | 6.60 | 1 | 1 | 0.3000 | 1.0740 | 1.9800 | 0.0011 | 3 | 5.9400 | 0.0032 | 5.9432 | 19.80 |
|  | Packaging | Paper | 0.0010 |  | 1 | 1 | 0.9200 | 0.1720 | 0.0009 | 0.0002 | 3 | 0.0028 | 0.0005 | 0.0033 | 0.00 |
|  | Packaging | LDPE | 0.0010 |  | 1 | 1 | 2.6000 | 0.1720 | 0.0026 | 0.0002 | 3 | 0.0078 | 0.0005 | 0.0083 | 0.00 |
| Biogel Surgeon Gloves 7.5 latex | Product | Rubber | 0.0070 | 1.06 | 1 | 1 | 0.3000 | 1.0740 | 0.3180 | 0.0075 | 3 | 0.9540 | 0.0226 | 0.9766 | 3.18 |
|  | Packaging | Paper | 0.0060 |  | 1 | 1 | 0.9200 | 0.1720 | 0.0055 | 0.0010 | 3 | 0.0166 | 0.0031 | 0.0197 | 0.00 |
|  | Packaging | LDPE | 0.0060 |  | 1 | 1 | 2.6000 | 0.1720 | 0.0156 | 0.0010 | 3 | 0.0468 | 0.0031 | 0.0499 | 0.00 |
| Gammex latex gloves 7 | Product | Latex | 0.0070 | 0.81 | 1 | 1 | 0.3000 | 1.0740 | 0.2430 | 0.0075 | 3 | 0.7290 | 0.0226 | 0.7516 | 2.43 |
|  | Packaging | Paper | 0.0040 |  | 1 | 1 | 0.9200 | 0.1720 | 0.0037 | 0.0007 | 3 | 0.0110 | 0.0021 | 0.0131 | 0.00 |
|  | Packaging | LDPE | 0.0030 |  | 1 | 1 | 2.6000 | 0.1720 | 0.0078 | 0.0005 | 3 | 0.0234 | 0.0015 | 0.0249 | 0.00 |
| Biogel Surgeon Gloves 6 | Product | Rubber | 0.0070 | 1.06 | 1 | 1 | 0.3000 | 1.0740 | 0.3180 | 0.0075 | 3 | 0.9540 | 0.0226 | 0.9766 | 3.18 |
|  | Packaging | Paper | 0.0060 |  | 1 | 1 | 0.9200 | 0.1720 | 0.0055 | 0.0010 | 3 | 0.0166 | 0.0031 | 0.0197 | 0.00 |
|  | Packaging | LDPE | 0.0060 |  | 1 | 1 | 2.6000 | 0.1720 | 0.0156 | 0.0010 | 3 | 0.0468 | 0.0031 | 0.0499 | 0.00 |
| Gammex latex gloves 6 | Product | Latex | 0.0070 | 0.81 | 1 | 1 | 0.3000 | 1.0740 | 0.2430 | 0.0075 | 3 | 0.7290 | 0.0226 | 0.7516 | 2.43 |
|  | Packaging | Paper | 0.0040 |  | 1 | 1 | 0.9200 | 0.1720 | 0.0037 | 0.0007 | 3 | 0.0110 | 0.0021 | 0.0131 | 0.00 |
|  | Packaging | LDPE | 0.0030 |  | 1 | 1 | 2.6000 | 0.1720 | 0.0078 | 0.0005 | 3 | 0.0234 | 0.0015 | 0.0249 | 0.00 |
| 500ml NaCL | Product | Pharmaceuticals | 0.5278 | 1.71 | 1 | 1 | 0.1550 |  | 0.0818 | 0.0000 | 3 | 0.2454 | 0.0000 | 0.2454 | 5.13 |
|  | Packaging | LDPE | 0.0250 |  | 1 | 1 | 2.6000 | 0.1720 | 0.0650 | 0.0043 | 3 | 0.1950 | 0.0129 | 0.2079 | 0.00 |
| Waste disposal bag | Product | LDPE | 0.0230 |  | 1 | 1 | 2.6000 | 1.0740 | 0.0598 | 0.0247 | 3 | 0.1794 | 0.0741 | 0.2535 | 0.00 |
| Surgical gown | Product | LDPE | 0.0350 |  | 1 | 1 | 2.6000 | 1.0740 | 0.0910 | 0.0376 | 8 | 0.7280 | 0.3007 | 1.0287 | 0.00 |
|  | Packaging | Paper | 0.0270 |  | 1 | 1 | 0.9200 | 0.1720 | 0.0248 | 0.0046 | 8 | 0.1987 | 0.0372 | 0.2359 | 0.00 |
|  | Packaging | LDPE | 0.0051 |  | 1 | 1 | 2.6000 | 0.1720 | 0.0133 | 0.0009 | 8 | 0.1061 | 0.0070 | 0.1131 | 0.00 |
| Small drape 75x75 | Product | Polypropylene | 0.0430 | 5.28 | 1 | 1 | 3.1050 | 1.0740 | 16.3944 | 0.0462 | 9 | 147.5496 | 0.4156 | 147.9652 | 47.52 |
|  | Packaging | Paper | 0.0010 |  | 1 | 1 | 0.9200 | 0.1720 | 0.0009 | 0.0002 | 9 | 0.0083 | 0.0015 | 0.0098 | 0.00 |
|  | Packaging | LDPE | 0.0100 |  | 1 | 1 | 2.6000 | 0.1720 | 0.0260 | 0.0017 | 9 | 0.2340 | 0.0155 | 0.2495 | 0.00 |
| Mayo stand cover 80X144 | Product | Polypropylene | 0.2090 | 1.11 | 1 | 1 | 3.1050 | 1.0740 | 3.4466 | 0.2245 | 9 | 31.0190 | 2.0202 | 33.0391 | 9.99 |
|  | Packaging | Paper | 0.0010 |  | 1 | 1 | 0.9200 | 0.1720 | 0.0009 | 0.0002 | 9 | 0.0083 | 0.0015 | 0.0098 | 0.00 |
|  | Packaging | LDPE | 0.0100 |  | 1 | 1 | 2.6000 | 0.1720 | 0.0260 | 0.0017 | 9 | 0.2340 | 0.0155 | 0.2495 | 0.00 |
| Trolley cover | Product | Polypropylene | 0.2040 | 2.83 | 1 | 1 | 3.1050 | 1.0740 | 8.7872 | 0.2191 | 9 | 79.0844 | 1.9719 | 81.0562 | 25.47 |
|  | Packaging | Paper | 0.0010 |  | 1 | 1 | 0.9200 | 0.1720 | 0.0009 | 0.0002 | 9 | 0.0083 | 0.0015 | 0.0098 | 0.00 |
|  | Packaging | LDPE | 0.0100 |  | 1 | 1 | 2.6000 | 0.1720 | 0.0260 | 0.0017 | 9 | 0.2340 | 0.0155 | 0.2495 | 0.00 |
| Drape pack  100x85 183x183 350x152 200x152 | Product | Polypropylene | 0.8790 | 11.40 | 1 | 1 | 3.1050 | 1.0740 | 2.7293 | 0.9440 | 9 | 24.5637 | 8.4964 | 33.0601 | 102.60 |
|  | Packaging | Paper | 0.0010 |  | 1 | 1 | 0.9200 | 0.1720 | 0.0009 | 0.0002 | 9 | 0.0083 | 0.0015 | 0.0098 | 0.00 |
|  | Packaging | LDPE | 0.0100 |  | 1 | 1 | 2.6000 | 0.1720 | 0.0260 | 0.0017 | 9 | 0.2340 | 0.0155 | 0.2495 | 0.00 |
| Vascular general set | Product | Stainless steel | 4.5600 |  | 2040 | 1 | 0.3700 | 0.5690 | 0.0008 | 2.5946 | 5 | 0.0041 | 12.9732 | 12.9773 | 0.00 |
| Suction connecting tube | Product | Polypropylene | 0.1114 | 0.98 | 1 | 1 | 0.3000 | 1.0740 | 0.2940 | 0.1196 | 8 | 2.3520 | 0.9571 | 3.3091 | 7.84 |
|  | Packaging | Paper | 0.0020 |  | 1 | 1 | 0.9190 | 0.1720 | 0.0018 | 0.0003 | 8 | 0.0147 | 0.0028 | 0.0175 | 0.00 |
|  | Packaging | LDPE | 0.0026 |  | 1 | 1 | 2.6000 | 0.1720 | 0.0068 | 0.0004 | 8 | 0.0541 | 0.0036 | 0.0577 | 0.00 |
| Temperature probe | Product | polyurethane | 0.0120 | 1.58 | 1 | 1 | 0.3000 | 1.0740 | 0.4740 | 0.0129 | 7 | 3.3180 | 0.0902 | 3.4082 | 11.06 |
|  | Packaging | Paper | 0.0016 |  | 1 | 1 | 0.9190 | 0.1720 | 0.0015 | 0.0003 | 7 | 0.0103 | 0.0019 | 0.0122 | 0.00 |
|  | Packaging | LDPE | 0.0014 |  | 1 | 1 | 2.6000 | 0.1720 | 0.0036 | 0.0002 | 7 | 0.0255 | 0.0017 | 0.0272 | 0.00 |
| Tegaderm 7cmx8xm | Product | LDPE | 0.0041 | 0.33 | 1 | 1 | 0.3000 | 1.0740 | 0.0990 | 0.0044 | 15 | 1.4850 | 0.0661 | 1.5511 | 4.95 |
|  | Packaging | LDPE | 0.0019 |  | 1 | 1 | 2.6000 | 0.1720 | 0.0049 | 0.0003 | 15 | 0.0741 | 0.0049 | 0.0790 | 0.00 |
| Anaesthesia face mask Pre-filled inflatable size 4 | Product | PVC | 0.0212 | 1.33 | 1 | 1 | 0.3000 | 1.0740 | 0.3990 | 0.0228 | 8 | 3.1920 | 0.1822 | 3.3742 | 10.64 |
|  | Packaging | LDPE | 0.0058 |  | 1 | 1 | 2.6000 | 0.1720 | 0.0151 | 0.0010 | 8 | 0.1206 | 0.0080 | 0.1286 | 0.00 |
| Laryngoscope (metal combi MAC 3m 110mm) | Product | Steel | 0.1350 | 3.60 | 1 | 1 | 0.3000 | 1.0740 | 1.0800 | 0.1450 | 7 | 7.5600 | 1.0149 | 8.5749 | 25.20 |
|  | Packaging | LDPE | 0.0010 |  | 1 | 1 | 2.6000 | 0.1720 | 0.0026 | 0.0002 | 7 | 0.0182 | 0.0012 | 0.0194 | 0.00 |
| Syringe 50ml | Product | Polypropylene | 0.0309 | 0.37 | 1 | 1 | 0.3000 | 1.0740 | 0.1110 | 0.0332 | 7 | 0.7770 | 0.2323 | 1.0093 | 2.59 |
|  | Packaging | Paper | 0.0015 |  | 1 | 1 | 0.9190 | 0.1720 | 0.0014 | 0.0003 | 7 | 0.0096 | 0.0018 | 0.0115 | 0.00 |
|  | Packaging | LDPE | 0.0036 |  | 1 | 1 | 2.6000 | 0.1720 | 0.0094 | 0.0006 | 7 | 0.0655 | 0.0043 | 0.0699 | 0.00 |
| Guedel Airway Size 4 | Product | LDPE | 0.0390 | 0.22 | 1 | 1 | 0.3000 | 1.0740 | 0.0660 | 0.0419 | 7 | 0.4620 | 0.2932 | 0.7552 | 1.54 |
|  | Packaging | LDPE | 0.0010 |  | 1 | 1 | 2.6000 | 0.1720 | 0.0026 | 0.0002 | 7 | 0.0182 | 0.0012 | 0.0194 | 0.00 |
| Syringe 5ml | Product | Polypropylene | 0.0044 | 0.07 | 1 | 1 | 0.3000 | 1.0740 | 0.0210 | 0.0047 | 10 | 0.2100 | 0.0473 | 0.2573 | 0.70 |
|  | Packaging | Paper | 0.0003 |  | 1 | 1 | 0.9190 | 0.1720 | 0.0003 | 0.0001 | 10 | 0.0028 | 0.0005 | 0.0033 | 0.00 |
|  | Packaging | LDPE | 0.0003 |  | 1 | 1 | 2.6000 | 0.1720 | 0.0008 | 0.0001 | 10 | 0.0078 | 0.0005 | 0.0083 | 0.00 |
| Syringe 2ml | Product | Polypropylene | 0.0025 | 0.04 | 1 | 1 | 0.3000 | 1.0740 | 0.0120 | 0.0027 | 9 | 0.1080 | 0.0242 | 0.1322 | 0.36 |
|  | Packaging | Paper | 0.0003 |  | 1 | 1 | 0.9190 | 0.1720 | 0.0003 | 0.0001 | 19 | 0.0052 | 0.0010 | 0.0062 | 0.00 |
|  | Packaging | LDPE | 0.0002 |  | 1 | 1 | 2.6000 | 0.1720 | 0.0005 | 0.0000 | 19 | 0.0099 | 0.0007 | 0.0105 | 0.00 |
| Syringe 10ml | Product | Polypropylene | 0.0068 | 0.10 | 1 | 1 | 0.3000 | 1.0740 | 0.0300 | 0.0073 | 13 | 0.3900 | 0.0949 | 0.4849 | 1.30 |
|  | Packaging | Paper | 0.0007 |  | 1 | 1 | 0.9190 | 0.1720 | 0.0006 | 0.0001 | 13 | 0.0084 | 0.0016 | 0.0099 | 0.00 |
|  | Packaging | LDPE | 0.0005 |  | 1 | 1 | 2.6000 | 0.1720 | 0.0013 | 0.0001 | 13 | 0.0169 | 0.0011 | 0.0180 | 0.00 |
| Syringe 20ml | Product | Polypropylene | 0.0129 | 0.11 | 1 | 1 | 0.3000 | 1.0740 | 0.0330 | 0.0139 | 19 | 0.6270 | 0.2632 | 0.8902 | 2.09 |
|  | Packaging | Paper | 0.0004 |  | 1 | 1 | 0.9190 | 0.1720 | 0.0004 | 0.0001 | 19 | 0.0070 | 0.0013 | 0.0083 | 0.00 |
|  | Packaging | LDPE | 0.0007 |  | 1 | 1 | 2.6000 | 0.1720 | 0.0018 | 0.0001 | 19 | 0.0346 | 0.0023 | 0.0369 | 0.00 |
| Sodium Chloride 0.9% 10ml | Product | Pharmaceuticals | 0.0125 | 0.21 | 1 | 1 | 0.3000 |  | 0.0630 |  | 19 | 1.1970 | 0.0000 | 1.1970 | 3.99 |
|  | Packaging | Polypropylene | 0.0015 |  | 1 | 1 | 3.1050 | 0.1720 | 0.0047 | 0.0003 | 19 | 0.0885 | 0.0049 | 0.0934 | 0.00 |
| Sodium Chloride 0.9% 5ml | Product | Pharmaceuticals | 0.0063 | 0.30 | 1 | 1 | 0.3000 |  | 0.0900 |  | 14 | 1.2600 | 0.0000 | 1.2600 | 4.20 |
|  | Packaging | Polypropylene | 0.0013 |  | 1 | 1 | 3.1050 | 0.1720 | 0.0040 | 0.0002 | 14 | 0.0565 | 0.0031 | 0.0596 | 0.00 |
| Optilube | Product | Pharmaceuticals | 0.0060 | 0.04 | 1 | 1 | 0.3000 |  | 0.0120 |  | 7 | 0.0840 | 0.0000 | 0.0840 | 0.28 |
|  | Packaging | Average plastics | 0.0040 |  | 1 | 1 | 3.1160 | 0.1720 | 0.0125 | 0.0007 | 7 | 0.0872 | 0.0048 | 0.0921 | 0.00 |
| Gloves | Product | Rubber | 0.0080 | 0.08 | 1 | 1 | 0.3000 | 1.0740 | 0.0240 | 0.0086 | 8 | 0.1920 | 0.0687 | 0.2607 | 0.64 |
| Water for injections 20ml | Product | Pharmaceuticals |  | 0.61 | 1 | 1 | 0.3000 |  | 0.1830 |  | 7 | 1.2810 | 0.0000 | 1.2810 | 4.27 |
|  | Packaging | PET | 0.0020 |  | 1 | 1 | 4.0320 | 0.1720 | 0.0081 | 0.0003 | 7 | 0.0564 | 0.0024 | 0.0589 | 0.00 |
| Box packaging 20 ampoules | Packaging | Cardboard | 0.0304 |  | 1 | 1 | 0.8840 | 0.1720 | 0.0269 | 0.0052 | 7 | 0.1881 | 0.0366 | 0.2247 | 0.00 |
| Propofol 200mg | Product | Pharmaceuticals |  | 0.96 | 1 | 1 | 0.1550 |  | 0.1488 |  | 9 | 1.0416 | 0.0000 | 1.0416 | 6.72 |
|  | Packaging | Glass | 0.1220 |  | 1 | 1 | 1.4020 | 1.0740 | 0.1710 | 0.1310 | 9 | 1.1973 | 0.9172 | 2.1145 | 0.00 |
| Ondansetron 4mg | Product | Pharmaceuticals | 0.0002 | 5.99 | 1 | 1 | 0.1550 |  | 0.9285 | 0.0000 | 8 | 7.4276 | 0.0000 | 7.4276 | 47.92 |
|  | Packaging | Glass | 0.0048 |  | 1 | 1 | 1.4020 | 1.0740 | 0.0067 | 0.0052 | 8 | 0.0538 | 0.0412 | 0.0951 | 0.00 |
| NaCl 1L | Product | Pharmaceuticals |  | 2.65 | 1 | 1 | 0.1550 |  | 0.4108 |  | 8 | 3.2860 | 0.0000 | 3.2860 | 21.20 |
|  | Packaging | LDPE | 0.0500 |  | 1 | 1 | 2.6010 | 0.1720 | 0.1301 | 0.0086 | 8 | 1.0404 | 0.0688 | 1.1092 | 0.00 |
| Sevoflurane 0.5 FiO2 | Product | Pharmaceuticals |  | 6.21 | 1 | 1 | 70.9400 |  | 70.9400 |  | 25.25 | 1791.2350 | 0.0000 | 1791.2350 | 156.80 |
| Fentanyl 50mcg/ml 2ml | Product | Pharmaceuticals | 0.0002 | 1.43 | 1 | 1 | 0.3000 |  | 0.4290 |  | 7 | 3.0030 | 0.0000 | 3.0030 | 10.01 |
|  | Packaging | Glass | 0.0048 |  | 1 | 1 | 1.4020 | 1.0740 | 0.0067 | 0.0052 | 7 | 0.0471 | 0.0361 | 0.0832 | 0.00 |
| Box packaging (10 ampoules) | Packaging | Cardboard | 0.0046 |  | 1 | 1 | 0.8840 | 0.1720 | 0.0041 | 0.0008 | 7 | 0.0285 | 0.0055 | 0.0340 | 0.00 |
|  | Packaging | LDPE | 0.0077 |  | 1 | 1 | 2.6000 | 0.1720 | 0.0200 | 0.0013 | 7 | 0.1401 | 0.0093 | 0.1494 | 0.00 |
| Ketamine 50mg | Product | Pharmaceuticals |  | 0.51 | 1 | 1 | 0.1550 |  | 0.0784 |  | 1 | 0.0784 | 0.0000 | 0.0784 | 0.51 |
|  | Packaging | Glass | 0.1220 |  | 1 | 1 | 1.4020 | 1.0740 | 0.1710 | 0.1310 | 1 | 0.1710 | 0.1310 | 0.3021 | 0.00 |
| Dexamethasone 6.6mg | Product | Pharmaceuticals |  | 2.44 | 1 | 1 | 0.1550 |  | 0.3788 | 0.0000 | 6 | 2.2729 | 0.0000 | 2.2729 | 14.64 |
|  | Packaging | Glass | 0.0048 |  | 1 | 1 | 1.4020 | 1.0740 | 0.0067 | 0.0052 | 6 | 0.0404 | 0.0309 | 0.0713 | 0.00 |
| neostigmine 2.5mg +Glycopyrronium 0.5mg /1ml | Product | Pharmaceuticals |  | 1.15 | 1 | 1 | 0.1550 |  | 0.1783 | 0.0000 | 5 | 0.8913 | 0.0000 | 0.8913 | 5.75 |
|  | Packaging | Glass | 0.0048 |  | 1 | 1 | 1.4020 | 1.0740 | 0.0067 | 0.0052 | 5 | 0.0336 | 0.0258 | 0.0594 | 0.00 |
| Metaraminol 0.5mg/1ml | Product | Pharmaceuticals |  | 0.75 | 1 | 1 | 0.1550 |  | 0.1159 | 0.0000 | 411 | 47.6513 | 0.0000 | 47.6513 | 308.25 |
|  | Packaging | Glass | 0.0010 |  | 1 | 1 | 1.4020 | 1.0740 | 0.0013 | 0.0010 | 411 | 0.5532 | 0.4238 | 0.9769 | 0.00 |
| Tracheal tube 7 | Product | Polyvinyl Chloride | 0.0614 | 0.96 | 1 | 1 | 0.3000 | 1.0740 | 0.2880 | 0.0659 | 4 | 1.1520 | 0.2638 | 1.4158 | 3.84 |
|  | Packaging | Paper | 0.0008 |  | 1 | 1 | 0.9190 | 0.1720 | 0.0007 | 0.0001 | 4 | 0.0029 | 0.0006 | 0.0035 | 0.00 |
|  | Packaging | LDPE | 0.0028 |  | 1 | 1 | 2.6000 | 0.1720 | 0.0073 | 0.0005 | 4 | 0.0291 | 0.0019 | 0.0310 | 0.00 |
| Large vascular set | Product | Stainless steel | 4.5600 |  | 2040 | 1 | 0.3700 | 0.5690 | 0.0008 | 2.5946 | 5 | 0.0041 | 12.9732 | 12.9773 | 0.00 |
| Small Vascular set | Product | Stainless steel | 3.7300 |  | 2040 | 1 | 0.3700 | 0.5690 | 0.0007 | 2.1224 | 4 | 0.0027 | 8.4895 | 8.4922 | 0.00 |
| Micro instruments | Product | Stainless steel | 1.4870 |  | 2040 | 1 | 0.3700 | 0.5690 | 0.0003 | 0.8461 | 4 | 0.0011 | 3.3844 | 3.3855 | 0.00 |
| Inadine 9.5cm x 9.5cm | Product | Polyethylene glycol | 0.0070 | 0.54 | 1 | 1 | 0.3000 | 1.0740 | 0.1620 | 0.0073 | 1 | 0.1620 | 0.0073 | 0.1693 | 0.54 |
|  | Packaging | Aluminium | 0.0030 |  | 1 | 1 | 9.1220 | 0.1720 | 0.0295 | 0.0006 | 1 | 0.0295 | 0.0006 | 0.0300 | 0.00 |
|  | Packaging | Cardboard | 0.0020 |  | 1 | 1 | 0.8840 | 0.1720 | 0.0016 | 0.0003 | 1 | 0.0016 | 0.0003 | 0.0020 | 0.00 |
| Kaltostat 15cm x 25cm | Product | Polysaccharide | 0.0070 | 4.80 | 1 | 1 | 0.3000 | 1.0740 | 1.4400 | 0.0073 | 1 | 1.4400 | 0.0073 | 1.4473 | 4.80 |
|  | Packaging | Aluminium | 0.0030 |  | 1 | 1 | 9.1220 | 0.1720 | 0.0295 | 0.0006 | 1 | 0.0295 | 0.0006 | 0.0300 | 0.00 |
|  | Packaging | Cardboard | 0.0020 |  | 1 | 1 | 0.8840 | 0.1720 | 0.0016 | 0.0003 | 1 | 0.0016 | 0.0003 | 0.0020 | 0.00 |
| Gauze Pack | Product | Cotton | 0.0050 | 1.70 | 1 | 1 | 0.3000 | 1.0740 | 0.0015 | 0.0055 | 3 | 0.0046 | 0.0165 | 0.0211 | 5.10 |
|  | Packaging | Paper | 0.0020 |  | 1 | 1 | 0.9190 | 0.1720 | 0.0020 | 0.0004 | 3 | 0.0061 | 0.0011 | 0.0072 | 0.00 |
| Wool bandage | Product | Wool | 0.0050 | 2.33 | 1 | 1 | 0.3000 | 1.0740 | 0.0015 | 0.0055 | 1 | 0.0015 | 0.0055 | 0.0070 | 2.33 |
|  | Packaging | Paper | 0.0020 |  | 1 | 1 | 0.9190 | 0.1720 | 0.0020 | 0.0004 | 1 | 0.0020 | 0.0004 | 0.0024 | 0.00 |
| Crepe bandage | Product | Cotton | 0.0050 | 0.55 | 1 | 1 | 0.3000 | 1.0740 | 0.0015 | 0.0055 | 2 | 0.0031 | 0.0110 | 0.0141 | 1.10 |
|  | Packaging | Paper | 0.0020 |  | 1 | 1 | 0.9190 | 0.1720 | 0.0020 | 0.0004 | 2 | 0.0041 | 0.0008 | 0.0048 | 0.00 |
| Atracurium 50mg/5ml (1mg) | Product | Pharmaceuticals |  | 0.06 | 1 | 1 | 0.1550 |  | 0.0093 |  | 290 | 2.7006 | 0.0000 | 2.7006 | 17.42 |
|  | Packaging | Glass | 0.0001 |  | 1 | 1 | 1.4020 | 1.0740 | 0.0002 | 0.0001 | 290 | 0.0529 | 0.0405 | 0.0933 | 0.00 |
| Box packaging 5 vials | Packaging | Cardboard | 0.0001 |  | 1 | 1 | 0.8840 | 0.1720 | 0.0001 | 0.0000 | 290 | 0.0251 | 0.0049 | 0.0300 | 0.00 |
|  | Packaging | Paper | 0.0002 |  | 1 | 1 | 0.9190 | 0.1720 | 0.0001 | 0.0000 | 290 | 0.0421 | 0.0079 | 0.0500 | 0.00 |
| Morphine 10mg/10ml (1ml) | Product | Pharmaceuticals |  | 0.18 | 1 | 1 | 0.1550 |  | 0.0271 | 0.0000 | 55 | 1.4919 | 0.0000 | 1.4919 | 9.63 |
|  | Packaging | Glass | 0.0005 |  | 1 | 1 | 1.4020 | 1.0740 | 0.0007 | 0.0005 | 55 | 0.0370 | 0.0284 | 0.0654 | 0.00 |
| Silicone catheter | Product | Rubber (silicone) | 0.0016 | 4.93 | 1 | 1 | 3.1800 | 1.0740 | 0.0051 | 0.0017 | 2 | 0.0102 | 0.0034 | 0.0136 | 9.86 |
|  | Packaging | LDPE | 0.0023 |  | 1 | 1 | 2.6010 | 0.1720 | 0.0060 | 0.0004 | 2 | 0.0120 | 0.0008 | 0.0128 | 0.00 |
|  | Packaging | Paper | 0.0010 |  | 1 | 1 | 0.9190 | 0.1720 | 0.0009 | 0.0002 | 2 | 0.0018 | 0.0003 | 0.0022 | 0.00 |
| Femoro-distal set | Product | Stainless steel | 2.9950 |  | 2040 | 1 | 0.3682 | 0.5690 | 0.0005 | 1.7042 | 4 | 0.0022 | 6.8166 | 6.8188 | 0.00 |
| LMA size 4 | Product | Polyvinyl Chloride | 0.0650 | 8.99 | 1 | 1 | 0.3000 | 1.0740 | 2.6970 | 0.0698 | 1 | 2.6970 | 0.0698 | 2.7668 | 8.99 |
|  | Packaging | Paper | 0.0080 |  | 1 | 1 | 0.9200 | 0.1720 | 0.0074 | 0.0014 | 1 | 0.0074 | 0.0014 | 0.0087 | 0.00 |
|  | Packaging | LDPE | 0.0280 |  | 1 | 1 | 2.6000 | 0.1720 | 0.0728 | 0.0048 | 1 | 0.0728 | 0.0048 | 0.0776 | 0.00 |
| Paracetamol 1g IV | Product | Pharmaceuticals |  | 1.35 | 1 | 1 | 0.1550 |  | 0.2093 |  | 1 | 0.2093 | 0.0000 | 0.2093 | 1.35 |
|  | Packaging | LDPE | 0.0240 |  | 1 | 1 | 2.6006 | 0.1720 | 0.0624 | 0.0041 | 1 | 0.0624 | 0.0041 | 0.0665 | 0.00 |
| tegaderm 8.5x11.5cm | Product | LPDE | 0.0060 | 1.14 | 1 | 1 | 0.3000 | 1.0740 | 0.3420 | 0.0064 | 2 | 0.6840 | 0.0129 | 0.6969 | 2.28 |
|  | Packaging | Paper | 0.0043 |  | 1 | 1 | 0.9190 | 0.1720 | 0.0040 | 0.0007 | 2 | 0.0079 | 0.0015 | 0.0094 | 0.00 |
|  | Packaging | LDPE | 0.0027 |  | 1 | 1 | 2.6000 | 0.1720 | 0.0070 | 0.0005 | 2 | 0.0140 | 0.0009 | 0.0150 | 0.00 |
| Ultrasound probe cover | Product | LDPE | 0.0872 | 4.13 | 1 | 1 | 0.3000 | 1.0740 | 1.2390 | 0.0937 | 2 | 2.4780 | 0.1873 | 2.6653 | 8.26 |
|  | Product | paper cover | 0.0045 |  | 1 | 1 | 0.9190 | 0.1720 | 0.0041 | 0.0008 | 2 | 0.0083 | 0.0015 | 0.0098 | 0.00 |
|  | Packaging | Paper | 0.0038 |  | 1 | 1 | 0.9190 | 0.1720 | 0.0035 | 0.0007 | 2 | 0.0070 | 0.0013 | 0.0083 | 0.00 |
|  | Packaging | LDPE | 0.0025 |  | 1 | 1 | 2.6000 | 0.1720 | 0.0065 | 0.0004 | 2 | 0.0130 | 0.0009 | 0.0139 | 0.00 |
| CVP line - 4 lumen catheter 8.5Fr/12.5cm | Product | Polyurethane | 0.0378 | 39.42 | 1 | 1 | 0.3000 | 1.0740 | 11.8260 | 0.0406 | 2 | 23.6520 | 0.0812 | 23.7332 | 78.84 |
|  | Packaging | Paper | 0.0032 |  | 1 | 1 | 0.9190 | 0.1720 | 0.0029 | 0.0006 | 2 | 0.0059 | 0.0011 | 0.0070 | 0.00 |
|  | Packaging | Polypropylene | 0.0400 |  | 1 | 1 | 3.1050 | 0.1720 | 0.1242 | 0.0069 | 2 | 0.2484 | 0.0138 | 0.2622 | 0.00 |
| Autofusion IV fluid infusion set 180cm tubing, 3 way connector, 120cm extension tubing, rotating luer connector | Product | Polypropylene | 0.0496 | 1.05 | 1 | 1 | 0.3000 | 1.0740 | 0.3150 | 0.0533 | 2 | 0.6300 | 0.1065 | 0.7365 | 2.10 |
|  | Packaging | Paper | 0.0007 |  | 1 | 1 | 0.9190 | 0.1720 | 0.0006 | 0.0001 | 2 | 0.0013 | 0.0002 | 0.0015 | 0.00 |
|  | Packaging | LDPE | 0.0007 |  | 1 | 1 | 2.6000 | 0.1720 | 0.0018 | 0.0001 | 2 | 0.0036 | 0.0002 | 0.0039 | 0.00 |
| Blue monofilament non-absorbable suture | Product | Steel + nylon | 0.0040 | 1.01 | 1 | 1 | 0.3000 | 1.0740 | 0.3030 | 0.0043 | 2 | 0.6060 | 0.0086 | 0.6146 | 2.02 |
|  | Packaging | Paper | 0.0005 |  | 1 | 1 | 0.9190 | 0.1720 | 0.0005 | 0.0001 | 2 | 0.0009 | 0.0002 | 0.0011 | 0.00 |
|  | Packaging | LDPE | 0.0005 |  | 1 | 1 | 2.6000 | 0.1720 | 0.0013 | 0.0001 | 2 | 0.0026 | 0.0002 | 0.0028 | 0.00 |
| Eco HEF + tube + connectors | Product | Polypropylene | 0.0317 | 1.23 | 1 | 1 | 0.3000 | 1.0740 | 0.3690 | 0.0340 | 2 | 0.7380 | 0.0681 | 0.8061 | 2.46 |
|  | Packaging | LDPE | 0.0003 |  | 1 | 1 | 2.6000 | 0.1720 | 0.0008 | 0.0001 | 2 | 0.0016 | 0.0001 | 0.0017 | 0.00 |
| Blunt Fill Needle with filter | Product | Steel | 0.0010 | 0.13 | 1 | 1 | 0.3000 | 1.0740 | 0.0390 | 0.0011 | 22 | 0.8580 | 0.0236 | 0.8816 | 2.86 |
|  | Packaging | Paper | 0.0003 |  | 1 | 1 | 0.9190 | 0.1720 | 0.0003 | 0.0001 | 22 | 0.0061 | 0.0011 | 0.0072 | 0.00 |
|  | Packaging | Pharmaceuticals | 0.0003 |  | 1 | 1 | 2.6000 | 0.1720 | 0.0008 | 0.0001 | 22 | 0.0172 | 0.0011 | 0.0183 | 0.00 |
| Lidocaine Hydrochloride 10mg/ml (1%) 5ml | Product | Pharmaceuticals | 0.0063 | 0.56 | 1 | 1 | 0.3000 |  | 0.1680 |  | 7 | 1.1760 | 0.0000 | 1.1760 | 3.92 |
|  | Packaging | Polypropylene | 0.0013 |  | 1 | 1 | 3.1050 | 0.1720 | 0.0040 | 0.0002 | 7 | 0.0283 | 0.0016 | 0.0298 | 0.00 |
| 0.9% NaCL 500ml | Product | Pharmaceuticals | 0.5278 | 1.71 | 1 | 1 | 0.3000 |  | 0.5130 |  | 6 | 3.0780 | 0.0000 | 3.0780 | 10.26 |
|  | Packaging | LDPE | 0.0200 |  | 1 | 1 | 2.6000 | 0.1720 | 0.0520 | 0.0034 | 6 | 0.3120 | 0.0206 | 0.3326 | 0.00 |
|  | Packaging | LDPE | 0.0102 |  | 1 | 1 | 2.6000 | 0.1720 | 0.0265 | 0.0018 | 6 | 0.1591 | 0.0105 | 0.1696 | 0.00 |
| Noradrenaline 4mg/50ml (1ml) | Product | Pharmaceuticals |  | 0.22 | 1 | 1 | 0.1550 |  | 0.0344 | 0.0000 | 175 | 6.0218 | 0.0000 | 6.0218 | 38.85 |
|  | Packaging | Glass | 0.0001 |  | 1 | 1 | 1.4020 | 1.0740 | 0.0001 | 0.0001 | 175 | 0.0236 | 0.0180 | 0.0416 | 0.00 |
| Magnesium 6mg | Product | Pharmaceuticals |  | 2.17 | 1 | 1 | 0.1550 |  | 0.3365 | 0.0000 | 3 | 1.0095 | 0.0000 | 1.0095 | 6.51 |
|  | Packaging | Glass | 0.0048 |  | 1 | 1 | 1.4020 | 1.0740 | 0.0067 | 0.0052 | 3 | 0.0202 | 0.0155 | 0.0357 | 0.00 |
| Heparin 1000u / 1ml | Product | Pharmaceuticals |  | 1.49 | 1 | 1 | 0.1550 |  | 0.2302 | 0.0000 | 19 | 4.3733 | 0.0000 | 4.3733 | 28.22 |
|  | Packaging | Glass | 0.0048 |  | 1 | 1 | 1.4020 | 1.0740 | 0.0067 | 0.0052 | 19 | 0.1279 | 0.0979 | 0.2258 | 0.00 |
| Hartmans 1L | Product | Pharmaceuticals |  | 2.21 | 1 | 1 | 0.1550 |  | 0.3426 |  | 8 | 2.7404 | 0.0000 | 2.7404 | 17.68 |
|  | Packaging | LDPE | 0.0500 |  | 1 | 1 | 2.6000 | 0.1720 | 0.1300 | 0.0086 | 8 | 1.0400 | 0.0688 | 1.1088 | 0.00 |
| Levobupivicaine 0.125% 25mg/20ml (1ml) | Product | Pharmaceuticals |  | 0.11 | 1 | 1 | 0.1550 |  | 0.0164 | 0.0000 | 120 | 1.9679 | 0.0000 | 1.9679 | 12.70 |
|  | Packaging | LDPE | 0.0002 |  | 1 | 1 | 1.4020 | 1.0740 | 0.0003 | 0.0003 | 120 | 0.0404 | 0.0309 | 0.0713 | 0.00 |
| Fistula set | Product | Stainless steel | 2.8000 |  | 2040 | 1 | 0.3682 | 0.5690 | 0.0005 | 1.5932 | 1 | 0.0005 | 1.5932 | 1.5937 | 0.00 |
| Sterile Biogel gloves x2 | Product | Rubber | 0.0180 | 1.06 | 1 | 1 | 3.1800 | 1.0740 | 3.3708 | 0.0193 | 4 | 13.4832 | 0.0773 | 13.5605 | 4.24 |
|  | Packaging | Paper | 0.0060 |  | 1 | 1 | 0.9200 | 0.1720 | 0.0055 | 0.0000 | 4 | 0.0221 | 0.0001 | 0.0222 | 0.00 |
|  | Packaging | LDPE | 0.0060 |  | 1 | 1 | 2.6000 | 0.1720 | 0.0156 | 0.0001 | 4 | 0.0624 | 0.0004 | 0.0628 | 0.00 |
| Chloraprep tint | Product | Polypropylene | 0.0120 | 1.10 | 1 | 1 | 3.1050 | 1.0740 | 3.4155 | 0.0129 | 3 | 10.2465 | 0.0387 | 10.2852 | 3.30 |
|  | Product | Chlorhexadine gluconate 2% w/v and isopropyl alcohol 70% v/v | 0.0140 |  | 1 | 1 | 0.1550 |  | 0.1705 |  | 3 | 0.5115 | 0.0000 | 0.5115 | 0.00 |
|  | Packaging | Paper | 0.0040 |  | 1 | 1 | 0.9200 | 0.1720 | 0.0037 | 0.0007 | 3 | 0.0110 | 0.0021 | 0.0131 | 0.00 |
|  | Packaging | LDPE | 0.0070 |  | 1 | 1 | 2.6000 | 0.1720 | 0.0182 | 0.0012 | 3 | 0.0546 | 0.0036 | 0.0582 | 0.00 |
| Ioban drapes 60 x 35cm | Product | LDPE | 0.5200 | 6.34 | 1 | 1 | 2.5742 | 1.0740 | 1.3386 | 0.5585 | 2 | 2.6771 | 1.1170 | 3.7941 | 12.68 |
|  | Packaging | LDPE | 0.0450 |  | 1 | 1 | 2.6006 | 0.1720 | 0.1170 | 0.0077 | 2 | 0.2341 | 0.0155 | 0.2495 | 0.00 |
|  | Packaging | Paper | 0.0280 |  | 1 | 1 | 0.9194 | 0.1720 | 0.0257 | 0.0048 | 2 | 0.0515 | 0.0096 | 0.0611 | 0.00 |
| 3/0 monocryl curve [3213H] | Product | Stainless steel | 0.0002 | 2.56 | 1 | 1 | 0.3683 | 1.0740 | 0.0001 | 0.0002 | 4 | 0.0003 | 0.0009 | 0.0012 | 10.24 |
|  | Packaging | LDPE | 0.0004 |  | 1 | 1 | 2.6010 | 0.1720 | 0.0010 | 0.0001 | 4 | 0.0042 | 0.0003 | 0.0044 | 0.00 |
|  | Packaging | Aluminium Foil | 0.0010 |  | 1 | 1 | 9.1220 | 0.1720 | 0.0091 | 0.0002 | 4 | 0.0365 | 0.0007 | 0.0372 | 0.00 |
| impra tunneler | Product | Stainless steel | 2.4948 |  | 2040 | 1 | 0.3680 | 0.5690 | 0.0005 | 1.4195 | 2 | 0.0009 | 2.8390 | 2.8399 | 0.00 |
| 10 beaver blade | Product | Stainless steel | 0.0004 | 3.12 | 1 | 1 | 0.3680 | 1.0740 | 0.0001 | 0.0004 | 3 | 0.0004 | 0.0013 | 0.0017 | 9.36 |
|  | Packaging | Aluminium Foil | 0.0005 |  | 1 | 1 | 9.1220 | 0.1720 | 0.0046 | 0.0001 | 3 | 0.0137 | 0.0003 | 0.0139 | 0.00 |
| 11 blade | Product | Stainless steel | 0.0004 | 3.12 | 1 | 1 | 0.3680 | 1.0740 | 0.0001 | 0.0004 | 2 | 0.0003 | 0.0009 | 0.0012 | 6.24 |
|  | Packaging | Aluminium Foil | 0.0005 |  | 1 | 1 | 9.1220 | 0.1720 | 0.0046 | 0.0001 | 2 | 0.0091 | 0.0002 | 0.0093 | 0.00 |
| 2.0 vicryl 320H | Product | Stainless steel | 0.0002 | 3.93 | 1 | 1 | 0.3680 | 1.0740 | 1.4462 | 0.0002 | 7 | 10.1237 | 0.0015 | 10.1252 | 27.51 |
|  | Packaging | LDPE | 0.0004 |  | 1 | 1 | 2.6010 | 0.1720 | 0.0010 | 0.0001 | 7 | 0.0073 | 0.0005 | 0.0078 | 0.00 |
|  | Packaging | Aluminium foil | 0.0010 |  | 1 | 1 | 9.1220 | 0.1720 | 0.0091 | 0.0002 | 7 | 0.0639 | 0.0012 | 0.0651 | 0.00 |
| 5.0 surgipro VP-705 | Product | Polypropylene | 0.0002 | 4.01 | 1 | 1 | 3.1050 | 1.0740 | 0.0006 | 0.0002 | 2 | 0.0012 | 0.0004 | 0.0017 | 8.02 |
|  | Packaging | LDPE | 0.0004 |  | 1 | 1 | 2.6010 | 0.1720 | 0.0010 | 0.0001 | 2 | 0.0021 | 0.0001 | 0.0022 | 0.00 |
|  | Packaging | LDPE | 0.0004 |  | 1 | 1 | 2.6010 | 0.1720 | 0.0010 | 0.0001 | 2 | 0.0021 | 0.0001 | 0.0022 | 0.00 |
| 7.0 Prolene 8735H | Product | Polypropylene | 0.0002 | 2.76 | 1 | 1 | 3.1050 | 1.0740 | 0.0006 | 0.0002 | 2 | 0.0012 | 0.0004 | 0.0017 | 5.52 |
|  | Packaging | LDPE | 0.0004 |  | 1 | 1 | 2.6010 | 0.1720 | 0.0010 | 0.0001 | 2 | 0.0021 | 0.0001 | 0.0022 | 0.00 |
|  | Packaging | LDPE | 0.0004 |  | 1 | 1 | 2.6010 | 0.1720 | 0.0010 | 0.0001 | 2 | 0.0021 | 0.0001 | 0.0022 | 0.00 |
| 6.0 prolene W8807 | Product | Polypropylene | 0.0002 | 2.76 | 1 | 1 | 3.1050 | 1.0740 | 0.0006 | 0.0002 | 2 | 0.0012 | 0.0004 | 0.0017 | 5.52 |
|  | Packaging | LDPE | 0.0004 |  | 1 | 1 | 2.6010 | 0.1720 | 0.0010 | 0.0001 | 2 | 0.0021 | 0.0001 | 0.0022 | 0.00 |
|  | Packaging | LDPE | 0.0004 |  | 1 | 1 | 2.6010 | 0.1720 | 0.0010 | 0.0001 | 2 | 0.0021 | 0.0001 | 0.0022 | 0.00 |
| Opsite large 10x30cm | Product | LDPE | 0.0096 | 0.42 | 1 | 1 | 2.6010 | 1.0740 | 0.0250 | 0.0103 | 2 | 0.0499 | 0.0206 | 0.0706 | 0.84 |
|  | Packaging | LDPE | 0.0012 |  | 1 | 1 | 2.6010 | 0.1720 | 0.0031 | 0.0002 | 2 | 0.0062 | 0.0004 | 0.0067 | 0.00 |
|  | Packaging | Paper | 0.0069 |  | 1 | 1 | 0.9190 | 0.1720 | 0.0063 | 0.0012 | 2 | 0.0127 | 0.0024 | 0.0151 | 0.00 |
| light handles | Product | LDPE | 0.0030 | 0.53 | 1 | 1 | 2.6010 | 1.0740 | 0.0078 | 0.0032 | 9 | 0.0702 | 0.0290 | 0.0992 | 4.77 |
|  | Product | PET (Foam) | 0.0020 |  | 1 | 1 | 4.0324 | 1.0740 | 0.0081 | 0.0021 | 9 | 0.0726 | 0.0193 | 0.0919 | 0.00 |
|  | Packaging | LDPE | 0.0035 |  | 1 | 1 | 2.6010 | 0.1720 | 0.0091 | 0.0006 | 9 | 0.0819 | 0.0054 | 0.0873 | 0.00 |
| diathermy + tip cleaner | Product | PVC | 0.0680 | 1.75 | 1 | 1 | 3.4130 | 1.0740 | 0.2321 | 0.0730 | 3 | 0.6963 | 0.2191 | 0.9153 | 5.25 |
|  | Packaging | Paper | 0.0045 |  | 1 | 1 | 0.9190 | 0.1720 | 0.0041 | 0.0008 | 3 | 0.0124 | 0.0023 | 0.0147 | 0.00 |
|  | Packaging | LDPE | 0.0040 |  | 1 | 1 | 2.6010 | 0.1720 | 0.0104 | 0.0007 | 3 | 0.0312 | 0.0021 | 0.0333 | 0.00 |
| skin marker pen | Product | LDPE | 0.0050 | 0.42 | 1 | 1 | 2.6010 | 1.0740 | 0.0130 | 0.0054 | 3 | 0.0390 | 0.0161 | 0.0551 | 1.26 |
|  | Packaging | LDPE | 0.0038 |  | 1 | 1 | 2.6010 | 0.1720 | 0.0099 | 0.0007 | 3 | 0.0297 | 0.0020 | 0.0316 | 0.00 |
|  | Packaging | Paper | 0.0012 |  | 1 | 1 | 0.9190 | 0.1720 | 0.0011 | 0.0002 | 3 | 0.0033 | 0.0006 | 0.0039 | 0.00 |
| magnetic discard a apd | Product | Rubber (silicone) | 0.8000 | 203.58 | 1036 | 1 | 3.1800 | 0.5690 | 0.0025 | 0.4552 | 3 | 0.0074 | 1.3656 | 1.3730 | 610.74 |
| Diathermy quiver | Product | Polypropylene | 0.0620 | 0.49 | 1 | 1 | 3.1050 | 1.0740 | 0.1925 | 0.0666 | 3 | 0.5775 | 0.1998 | 0.7773 | 1.47 |
|  | Packaging | LDPE | 0.0045 |  | 1 | 1 | 2.6010 | 0.1720 | 0.0117 | 0.0008 | 3 | 0.0351 | 0.0023 | 0.0374 | 0.00 |
|  | Packaging | Paper | 0.0025 |  | 1 | 1 | 0.9190 | 0.1720 | 0.0023 | 0.0004 | 3 | 0.0069 | 0.0013 | 0.0082 | 0.00 |
| small fogarty inserts | Product | rubber | 0.0040 | 11.50 | 1 | 1 | 3.1800 | 1.0740 | 0.0127 | 0.0043 | 2 | 0.0254 | 0.0086 | 0.0340 | 23.00 |
|  | Packaging | Paper | 0.0010 |  | 1 | 1 | 0.9190 | 0.1720 | 0.0009 | 0.0002 | 2 | 0.0018 | 0.0003 | 0.0022 | 0.00 |
|  | Packaging | LDPE | 0.0015 |  | 1 | 1 | 2.6010 | 0.1720 | 0.0039 | 0.0003 | 2 | 0.0078 | 0.0005 | 0.0083 | 0.00 |
| Vascular shods | Product | Rubber | 0.0010 | 2.02 | 1 | 1 | 3.1800 | 1.0740 | 0.0032 | 0.0011 | 2 | 0.0064 | 0.0021 | 0.0085 | 4.04 |
|  | Product | PET (Foam) | 0.0020 |  | 1 | 1 | 4.0320 | 1.0740 | 0.0081 | 0.0021 | 2 | 0.0161 | 0.0043 | 0.0204 | 0.00 |
|  | Packaging | LDPE | 0.0015 |  | 1 | 1 | 2.6010 | 0.1720 | 0.0039 | 0.0003 | 2 | 0.0078 | 0.0005 | 0.0083 | 0.00 |
|  | Packaging | Paper | 0.0010 |  | 1 | 1 | 0.9190 | 0.1720 | 0.0009 | 0.0002 | 2 | 0.0018 | 0.0003 | 0.0022 | 0.00 |
| Gallipots | Product | Polypropylene | 0.0190 | 0.10 | 1 | 1 | 3.1050 | 1.0740 | 0.0590 | 0.0204 | 9 | 0.5310 | 0.1837 | 0.7146 | 0.90 |
|  | Packaging | LDPE | 0.0018 |  | 1 | 1 | 2.6010 | 0.1720 | 0.0047 | 0.0003 | 9 | 0.0421 | 0.0028 | 0.0449 | 0.00 |
|  | Packaging | Paper | 0.0012 |  | 1 | 1 | 0.9190 | 0.1720 | 0.0011 | 0.0002 | 9 | 0.0099 | 0.0019 | 0.0118 | 0.00 |
| Scanlan Tunneler Sheath | Product | Rubber (silicone) | 0.0652 | 18.38 | 1 | 1 | 3.1800 | 1.0740 | 0.2073 | 0.0700 | 2 | 0.4147 | 0.1400 | 0.5547 | 36.76 |
|  | Packaging | LDPE | 0.0440 |  | 1 | 1 | 2.6010 | 0.1720 | 0.1144 | 0.0076 | 2 | 0.2289 | 0.0151 | 0.2440 | 0.00 |
|  | Packaging | Paper | 0.0240 |  | 1 | 1 | 0.9190 | 0.1720 | 0.0221 | 0.0041 | 2 | 0.0441 | 0.0083 | 0.0524 | 0.00 |
| aldon bag | Product | PVC | 0.0650 | 1.24 | 1 | 1 | 3.4130 | 1.0740 | 0.2218 | 0.0698 | 3 | 0.6655 | 0.2094 | 0.8750 | 3.72 |
|  | Packaging | LDPE | 0.0420 |  | 1 | 1 | 2.6010 | 0.1720 | 0.1092 | 0.0072 | 3 | 0.3277 | 0.0217 | 0.3494 | 0.00 |
|  | Packaging | Paper | 0.0230 |  | 1 | 1 | 0.9190 | 0.1720 | 0.0211 | 0.0040 | 3 | 0.0634 | 0.0119 | 0.0753 | 0.00 |
| Foley catheter | Product | Rubber (silicone) | 0.0016 | 3.09 | 1 | 1 | 3.1800 | 1.0740 | 0.0051 | 0.0017 | 1 | 0.0051 | 0.0017 | 0.0068 | 3.09 |
|  | Packaging | LDPE | 0.0023 |  | 1 | 1 | 2.6010 | 0.1720 | 0.0060 | 0.0004 | 1 | 0.0060 | 0.0004 | 0.0064 | 0.00 |
|  | Packaging | Paper | 0.0010 |  | 1 | 1 | 0.9190 | 0.1720 | 0.0009 | 0.0002 | 1 | 0.0009 | 0.0002 | 0.0011 | 0.00 |
| Bard Hickman catheter Silicone 10.8 | Product | Silicone | 0.0740 | 201.60 | 1 | 1 | 0.3000 | 1.0740 |  |  | 1 | 0.0000 | 0.0000 | 0.0000 | 201.60 |
|  | Packaging | LDPE | 0.0040 |  | 1 | 1 |  | 0.1720 |  |  | 1 | 0.0000 | 0.0000 | 0.0000 | 0.00 |
| Softpore 5cm x 25cm | Product | LDPE | 0.0110 | 0.16 | 1 | 1 | 0.3000 | 1.0740 | 0.0480 | 0.1718 | 1 | 0.0480 | 0.1718 | 0.2198 | 0.16 |
|  | Packaging | Paper | 0.0040 |  | 1 | 1 | 0.9190 | 0.1720 | 0.0037 | 0.0007 | 1 | 0.0037 | 0.0007 | 0.0044 | 0.00 |
| Glycopyrronium Bromide 200mcg/ml - 1ml | Product | Pharmaceuticals |  | 1.65 | 1 | 1 | 0.3000 |  | 0.4950 |  | 1 | 0.4950 | 0.0000 | 0.4950 | 1.65 |
|  | Packaging | Glass | 0.0030 |  | 1 | 1 | 1.4020 | 1.0740 | 0.0042 | 0.0032 | 1 | 0.0042 | 0.0032 | 0.0074 | 0.00 |
| Plastic blister pack (5 ampoules) |  | PET | 0.0010 |  | 1 | 1 | 4.0320 | 0.1720 | 0.0040 | 0.0002 | 1 | 0.0040 | 0.0002 | 0.0042 | 0.00 |
| Box packaging (10 ampoules) | Packaging | Cardboard | 0.0079 |  | 1 | 1 | 0.8840 | 0.1720 | 0.0070 | 0.0014 | 1 | 0.0070 | 0.0014 | 0.0083 | 0.00 |
|  | Packaging | LDPE | 0.0040 |  | 1 | 1 | 2.6000 | 0.1720 | 0.0104 | 0.0007 | 1 | 0.0104 | 0.0007 | 0.0111 | 0.00 |
| Dexamethasone 3.3mg | Product | Pharmaceuticals |  | 2.40 | 1 | 1 | 0.1550 |  | 0.3718 | 0.0000 | 4 | 1.4874 | 0.0000 | 1.4874 | 9.60 |
|  | Packaging | Glass | 0.0048 |  | 1 | 1 | 1.4020 | 1.0740 | 0.0067 | 0.0052 | 4 | 0.0269 | 0.0206 | 0.0475 | 0.00 |
| Ephedrine 30mg/10ml | Product | Pharmaceuticals |  | 12.41 | 1 | 1 | 0.1550 |  | 1.9234 | 0.0000 | 2 | 3.8468 | 0.0000 | 3.8468 | 24.82 |
|  | Packaging | Glass | 0.0048 |  | 1 | 1 | 1.4020 | 1.0740 | 0.0067 | 0.0052 | 2 | 0.0135 | 0.0103 | 0.0238 | 0.00 |
| Mixed retractors set | Product | Stainless steel | 4.6500 |  | 2040 | 1 | 0.3682 | 0.5690 | 0.0000 | 0.0000 | 1 | 0.0000 | 0.0000 | 0.0000 | 0.00 |
| 6x4 swab pack | Product | Cotton | 0.0800 | 4.03 | 1 | 1 | 0.3000 | 1.0740 | 1.2090 | 0.0859 | 6 | 7.2540 | 0.5155 | 7.7695 | 24.18 |
|  | Packaging | Paper | 0.0045 |  | 1 | 1 | 0.9190 | 0.1720 | 0.0041 | 0.0008 | 6 | 0.0248 | 0.0046 | 0.0295 | 0.00 |
| Rocuronium bromide 50mg/5ml | Product | Pharmaceuticals |  | 2.80 | 1 | 1 | 0.3000 |  | 0.8400 |  | 1 | 0.8400 | 0.0000 | 0.8400 | 2.80 |
|  | Packaging | Glass | 0.0030 |  | 1 | 1 | 1.4020 | 1.0740 | 0.0042 | 0.0032 | 1 | 0.0042 | 0.0032 | 0.0074 | 0.00 |
| Plastic blister pack (5 ampoules) | Packaging | PET | 0.0010 |  | 1 | 1 | 4.0320 | 0.1720 | 0.0040 | 0.0002 | 1 | 0.0040 | 0.0002 | 0.0042 | 0.00 |
| Box packaging (10 ampoules) | Packaging | Cardboard | 0.0079 |  | 1 | 1 | 0.8840 | 0.1720 | 0.0070 | 0.0014 | 1 | 0.0070 | 0.0014 | 0.0083 | 0.00 |
|  | Packaging | LDPE | 0.0040 |  | 1 | 1 | 2.6000 | 0.1720 | 0.0104 | 0.0007 | 1 | 0.0104 | 0.0007 | 0.0111 | 0.00 |
| Levobupivicaine 25mg/10ml | Product | Pharmaceuticals | 0.0002 | 2.12 | 1 | 1 | 0.3000 |  | 0.6348 |  | 1 | 0.6348 | 0.0000 | 0.6348 | 2.12 |
|  | Packaging | Glass | 0.0048 |  | 1 | 1 | 1.4020 | 1.0740 | 0.0067 | 0.0052 | 1 | 0.0067 | 0.0052 | 0.0119 | 0.00 |
| Box packaging (10 ampoules) | Packaging | Cardboard | 0.0046 |  | 1 | 1 | 0.8840 | 0.1720 | 0.0041 | 0.0008 | 1 | 0.0041 | 0.0008 | 0.0049 | 0.00 |
|  | Packaging | LDPE | 0.0077 |  | 1 | 1 | 2.6000 | 0.1720 | 0.0200 | 0.0013 | 1 | 0.0200 | 0.0013 | 0.0213 | 0.00 |
| Cyclizine 50mg/1ml | Product | Pharmaceuticals | 0.0005 | 2.18 | 1 | 1 | 0.1550 |  | 0.3376 |  | 1 | 0.3376 | 0.0000 | 0.3376 | 2.18 |
|  | Packaging | Glass | 0.0065 |  | 1 | 1 | 1.4020 | 1.0740 | 0.0091 | 0.0070 | 1 | 0.0091 | 0.0070 | 0.0161 | 0.00 |
| Box packaging 5 vials | Packaging | Cardboard | 0.0049 |  | 1 | 1 | 0.8840 | 0.1720 | 0.0043 | 0.0008 | 1 | 0.0043 | 0.0008 | 0.0052 | 0.00 |
|  | Packaging | Paper | 0.0079 |  | 1 | 1 | 0.9190 | 0.1720 | 0.0073 | 0.0014 | 1 | 0.0073 | 0.0014 | 0.0086 | 0.00 |
| Amputation set | Product | Stainless steel | 4.7830 |  | 2040 | 1 | 0.3700 | 0.5690 | 0.0009 | 2.7215 | 1 | 0.0009 | 2.7215 | 2.7224 | 0.00 |
| Desouter set | Product | Stainless steel | 4.0710 |  | 2040 | 1 | 0.3700 | 0.5690 | 0.0007 | 2.3164 | 1 | 0.0007 | 2.3164 | 2.3171 | 0.00 |
| 18x18 swab pack | Product | Cotton | 0.0800 | 1.29 | 1 | 1 | 0.3000 | 1.0740 | 0.3870 | 0.0859 | 1 | 0.3870 | 0.0859 | 0.4729 | 1.29 |
|  | Packaging | Paper | 0.0045 |  | 1 | 1 | 0.9190 | 0.1720 | 0.0041 | 0.0008 | 1 | 0.0041 | 0.0008 | 0.0049 | 0.00 |
| Aquacel ribbon 2cm x 45cm | Product | Polyurethane | 0.0117 | 1.67 | 1 | 1 | 0.3000 | 1.0740 | 0.5010 | 0.0125 | 3 | 1.5030 | 0.0375 | 1.5405 | 5.01 |
|  | Packaging | Paper | 0.0069 |  | 1 | 1 | 0.9190 | 0.1720 | 0.0063 | 0.0012 | 3 | 0.0190 | 0.0036 | 0.0226 | 0.00 |
|  | Packaging | Cardboard | 0.0042 |  | 1 | 1 | 0.8840 | 0.1720 | 0.0037 | 0.0007 | 3 | 0.0111 | 0.0022 | 0.0132 | 0.00 |
| Midazolam 2m/2ml (1ml) | Product | Pharmaceuticals |  | 0.47 | 1 | 1 | 0.1550 |  | 0.0721 | 0.0000 | 2 | 0.1442 | 0.0000 | 0.1442 | 0.93 |
|  | Packaging | Glass | 0.0005 |  | 1 | 1 | 1.4020 | 1.0740 | 0.0007 | 0.0005 | 2 | 0.0013 | 0.0010 | 0.0024 | 0.00 |
| Lignocaine 1% 100mg/10ml | Product | Pharmaceuticals |  | 0.63 | 1 | 1 | 0.1550 |  | 0.0978 | 0.0000 | 1 | 0.0978 | 0.0000 | 0.0978 | 0.63 |
|  | Packaging | LDPE | 0.0240 |  | 1 | 1 | 2.6006 | 0.1720 | 0.0624 | 0.0041 | 1 | 0.0624 | 0.0041 | 0.0665 | 0.00 |

| Dressings | | | | | | | | | | | | | | | |
| --- | --- | --- | --- | --- | --- | --- | --- | --- | --- | --- | --- | --- | --- | --- | --- |
| Product | Component | Material | Weight (kg) | Cost (£) | Number of uses | Number of scenarios per use | Emissions factor | Waste stream Emission Factor | Production | Waste | Number | Production total | Waste total | Total emissions | Total Cost (£) |
| Inadine 5cm x 5cm | Product | Polyethylene | 0.0019 | 0.37 | 1 | 1 | 0.3000 | 1.0740 | 0.1110 | 0.0021 | 10 | 1.1100 | 0.0207 | 1.1307 | 3.70 |
|  | Packaging | Aluminium | 0.0027 |  | 1 | 1 | 9.1220 | 0.1720 | 0.0244 | 0.0005 | 10 | 0.2444 | 0.0046 | 0.2490 | 0.00 |
|  | Packaging | Cardboard | 0.0007 |  | 1 | 1 | 0.8840 | 0.1720 | 0.0006 | 0.0001 | 10 | 0.0059 | 0.0011 | 0.0071 | 0.00 |
| Inadine 9.5cm x 9.5cm | Product | Polyethylene | 0.0068 | 0.54 | 1 | 1 | 0.3000 | 1.0740 | 0.1620 | 0.0073 | 17 | 2.7540 | 0.1237 | 2.8777 | 9.18 |
|  | Packaging | Aluminium | 0.0032 |  | 1 | 1 | 9.1220 | 0.1720 | 0.0295 | 0.0006 | 17 | 0.5012 | 0.0095 | 0.5106 | 0.00 |
|  | Packaging | Cardboard | 0.0019 |  | 1 | 1 | 0.8840 | 0.1720 | 0.0016 | 0.0003 | 17 | 0.0280 | 0.0054 | 0.0335 | 0.00 |
| Softpore 5cm x 15cm PO | Product | Polyethylene | 0.0090 | 0.12 | 1 | 1 | 0.3000 | 1.0740 | 0.0360 | 0.0097 | 10 | 0.3600 | 0.0967 | 0.4567 | 1.20 |
|  | Packaging | Paper | 0.0045 |  | 1 | 1 | 0.9190 | 0.1720 | 0.0041 | 0.0008 | 10 | 0.0409 | 0.0077 | 0.0486 | 0.00 |
| Softpore 5cm x 25cm | Product | Polyethylene | 0.0110 | 0.16 | 1 | 1 | 0.3000 | 1.0740 | 0.0480 | 0.1718 | 11 | 0.5280 | 1.8902 | 2.4182 | 1.76 |
|  | Packaging | Paper | 0.0040 |  | 1 | 1 | 0.9190 | 0.1720 | 0.0037 | 0.0007 | 11 | 0.0404 | 0.0076 | 0.0480 | 0.00 |
| Softpore 10cm x 15cm | Product | Polyethylene | 0.0017 | 0.09 | 1 | 1 | 0.3000 | 1.0740 | 0.0270 | 0.0018 | 17 | 0.4590 | 0.0307 | 0.4897 | 1.53 |
|  | Packaging | Paper | 0.0045 |  | 1 | 1 | 0.9190 | 0.1720 | 0.0041 | 0.0008 | 17 | 0.0695 | 0.0130 | 0.0826 | 0.00 |
| Softpore 10cm x 30cm | Product | Polyethylene | 0.0246 | 0.19 | 1 | 1 | 0.3000 | 1.0740 | 0.0570 | 0.0264 | 47 | 2.6790 | 1.2426 | 3.9216 | 8.93 |
|  | Packaging | Paper | 0.0019 |  | 1 | 1 | 0.9190 | 0.1720 | 0.0018 | 0.0003 | 47 | 0.0830 | 0.0155 | 0.0986 | 0.00 |
| Softpore 10cm x 35cm | Product | Polyethylene | 0.0190 | 0.27 | 1 | 1 | 0.3000 | 1.0740 | 0.0810 | 0.0204 | 10 | 0.8100 | 0.2041 | 1.0141 | 2.70 |
|  | Packaging | Paper | 0.0060 |  | 1 | 1 | 0.9190 | 0.1720 | 0.0055 | 0.0010 | 10 | 0.0551 | 0.0103 | 0.0655 | 0.00 |
| Mepore 11cm x 15cm | Product | Polyethylene | 0.0015 | 0.97 | 1 | 1 | 0.3000 | 1.0740 | 0.2910 | 0.0016 | 6 | 1.7460 | 0.0096 | 1.7556 | 5.82 |
|  | Packaging | LDPE | 0.0039 |  | 1 | 1 | 2.6006 | 0.1720 | 0.0103 | 0.0007 | 6 | 0.0616 | 0.0041 | 0.0656 | 0.00 |
|  | Packaging | Cardboard | 0.0025 |  | 1 | 1 | 0.8840 | 0.1720 | 0.0022 | 0.0004 | 6 | 0.0131 | 0.0025 | 0.0156 | 0.00 |
| Opsite large 10x30cm | Product | LDPE | 0.0096 | 0.42 | 1 | 1 | 2.6010 | 1.0740 | 0.0250 | 0.0103 | 2 | 0.0499 | 0.0206 | 0.0706 | 0.84 |
|  | Packaging | LDPE | 0.0012 |  | 1 | 1 | 2.6010 | 0.1720 | 0.0031 | 0.0002 | 2 | 0.0062 | 0.0004 | 0.0067 | 0.00 |
|  | Packaging | Paper | 0.0069 |  | 1 | 1 | 0.9190 | 0.1720 | 0.0063 | 0.0012 | 2 | 0.0127 | 0.0024 | 0.0151 | 0.00 |
| Mepilex border dressing 10cm x 12.5cm | Product | Polyurethane | 0.0085 | 3.17 | 1 | 1 | 0.3000 | 1.0740 | 0.9510 | 0.0091 | 36 | 34.2360 | 0.3272 | 34.5632 | 114.12 |
|  | Packaging | Paper | 0.0064 |  | 1 | 1 | 0.9190 | 0.1720 | 0.0059 | 0.0011 | 36 | 0.2118 | 0.0396 | 0.2514 | 0.00 |
|  | Packaging | Cardboard | 0.0076 |  | 1 | 1 | 0.8840 | 0.1720 | 0.0067 | 0.0013 | 36 | 0.2425 | 0.0472 | 0.2897 | 0.00 |
| Allevyn GB lite 5cm x 5cm | Product | Polyurethane | 0.0011 | 0.60 | 1 | 1 | 0.3000 | 1.0740 | 0.1800 | 0.0012 | 2 | 0.3600 | 0.0024 | 0.3624 | 1.20 |
|  | Packaging | Paper | 0.0009 |  | 1 | 1 | 0.9190 | 0.1720 | 0.0008 | 0.0002 | 2 | 0.0016 | 0.0003 | 0.0019 | 0.00 |
|  | Packaging | Cardboard | 0.0009 |  | 1 | 1 | 0.8830 | 0.1720 | 0.0008 | 0.0002 | 2 | 0.0016 | 0.0003 | 0.0020 | 0.00 |
| Allevyn GB 10cm x 10cm | Product | Polyurethane | 0.0117 | 1.29 | 1 | 1 | 0.3000 | 1.0740 | 0.3870 | 0.0125 | 30 | 11.6100 | 0.3754 | 11.9854 | 38.70 |
|  | Packaging | Paper | 0.0069 |  | 1 | 1 | 0.9190 | 0.1720 | 0.0063 | 0.0012 | 30 | 0.1903 | 0.0356 | 0.2259 | 0.00 |
|  | Packaging | Cardboard | 0.0036 |  | 1 | 1 | 0.8840 | 0.1720 | 0.0032 | 0.0006 | 30 | 0.0948 | 0.0006 | 0.0954 | 0.00 |
| Allevyn GB 10cm x 20cm | Product | Polyurethane | 0.0117 | 2.10 | 1 | 1 | 0.3000 | 1.0740 | 0.6300 | 0.0125 | 15 | 9.4500 | 0.1877 | 9.6377 | 31.50 |
|  | Packaging | Paper | 0.0069 |  | 1 | 1 | 0.9190 | 0.1720 | 0.0063 | 0.0012 | 15 | 0.0951 | 0.0178 | 0.1129 | 0.00 |
|  | Packaging | Cardboard | 0.0036 |  | 1 | 1 | 0.8840 | 0.1720 | 0.0032 | 0.0006 | 15 | 0.0474 | 0.0006 | 0.0480 | 0.00 |
| Allevyn foam 10cm x 20cm | Product | Polyurethane | 0.0180 | 2.62 | 1 | 1 | 0.3000 | 1.0740 | 0.7860 | 0.0193 | 5 | 3.9300 | 0.0967 | 4.0267 | 13.10 |
|  | Packaging | Paper | 0.0060 |  | 1 | 1 | 0.9190 | 0.1720 | 0.0055 | 0.0010 | 5 | 0.0276 | 0.0052 | 0.0327 | 0.00 |
| Allevyn Classic 12.5cm x 12.5cm | Product | Polyurethane | 0.0044 | 0.80 | 1 | 1 | 0.3000 | 1.0740 | 0.2400 | 0.0047 | 17 | 4.0800 | 0.0047 | 4.0847 | 13.60 |
|  | Packaging | Paper | 0.0042 |  | 1 | 1 | 0.9190 | 0.1720 | 0.0039 | 0.0007 | 17 | 0.0656 | 0.0007 | 0.0664 | 0.00 |
|  | Packaging | Cardboard | 0.0036 |  | 1 | 1 | 0.8840 | 0.1720 | 0.0032 | 0.0006 | 17 | 0.0537 | 0.0006 | 0.0543 | 0.00 |
| UrgoTul 10cm x 10cm | Product | Polyester | 0.0021 | 1.88 | 1 | 1 | 0.1550 | 1.0740 | 0.0003 | 0.0022 | 2 | 0.0006 | 0.0044 | 0.0051 | 3.76 |
|  | Packaging | Aluminium | 0.0040 |  | 1 | 1 | 9.1220 | 0.1720 | 0.0363 | 0.0007 | 2 | 0.0726 | 0.0014 | 0.0739 | 0.00 |
|  | Packaging | Cardboard | 0.0019 |  | 1 | 1 | 0.8840 | 0.1720 | 0.0017 | 0.0003 | 2 | 0.0034 | 0.0007 | 0.0041 | 0.00 |
| Leukomed 10cm x 20cm | Product | Polyurethane | 0.0230 | 8.78 | 1 | 1 | 0.3000 | 1.0740 | 2.6340 | 0.0247 | 1 | 2.6340 | 0.0247 | 2.6587 | 8.78 |
|  | Packaging | Paper | 0.0090 |  | 1 | 1 | 0.9190 | 0.1720 | 0.0083 | 0.0015 | 1 | 0.0083 | 0.0015 | 0.0098 | 0.00 |
| tegaderm 8.5x11.5cm | Product | LDPE | 0.0060 | 1.14 | 1 | 1 | 0.3000 | 1.0740 | 0.3420 | 0.0064 | 10 | 3.4200 | 0.0644 | 3.4844 | 11.40 |
|  | Packaging | Paper | 0.0043 |  | 1 | 1 | 0.9190 | 0.1720 | 0.0040 | 0.0007 | 10 | 0.0395 | 0.0074 | 0.0469 | 0.00 |
|  | Packaging | LDPE | 0.0027 |  | 1 | 1 | 2.6000 | 0.1720 | 0.0070 | 0.0005 | 10 | 0.0702 | 0.0046 | 0.0748 | 0.00 |
| Aquacell Ag 10cm x 10cm | Product | Polyurethane | 0.0017 | 3.45 | 1 | 1 | 0.3000 | 1.0740 | 0.0000 | 0.0018 | 26 | 0.0000 | 0.0469 | 0.0469 | 0.00 |
|  | Packaging | Aluminium | 0.0045 |  | 1 | 1 | 9.1220 | 0.1720 | 0.0406 | 0.0008 | 26 | 1.0557 | 0.0199 | 1.0756 | 0.00 |
|  | Packaging | Cardboard | 0.0025 |  | 1 | 1 | 0.8840 | 0.1720 | 0.0022 | 0.0004 | 26 | 0.0564 | 0.0110 | 0.0674 | 0.00 |
| Aquacell extra 10cm x 10cm | Product | Polyurethane | 0.0015 | 1.67 | 1 | 1 | 0.3000 | 1.0740 | 0.0000 | 0.0016 | 10 | 0.0000 | 0.0159 | 0.0159 | 0.00 |
|  | Packaging | LDPE | 0.0039 |  | 1 | 1 | 2.6006 | 0.1720 | 0.0103 | 0.0007 | 10 | 0.1026 | 0.0068 | 0.1094 | 0.00 |
|  | Packaging | Cardboard | 0.0025 |  | 1 | 1 | 0.8840 | 0.1720 | 0.0022 | 0.0004 | 10 | 0.0218 | 0.0042 | 0.0260 | 0.00 |
| Atraumen 5cm x 5cm | Product | Polyethylene | 0.0007 | 0.22 | 1 | 1 | 0.1550 | 1.0740 | 0.0001 | 0.0008 | 1 | 0.0001 | 0.0008 | 0.0009 | 0.22 |
|  | Packaging | Aluminium | 0.0022 |  | 1 | 1 | 9.1220 | 0.1720 | 0.0198 | 0.0004 | 1 | 0.0198 | 0.0004 | 0.0202 | 0.00 |
|  | Packaging | Cardboard | 0.0004 |  | 1 | 1 | 0.8840 | 0.1720 | 0.0003 | 0.0001 | 1 | 0.0003 | 0.0001 | 0.0004 | 0.00 |
| Atraumen 10cm x 20cm | Product | Polyethylene | 0.0045 | 0.38 | 1 | 1 | 0.3000 | 1.0740 | 0.1140 | 0.0049 | 5 | 0.5700 | 0.0243 | 0.5943 | 1.90 |
|  | Packaging | Aluminium | 0.0094 |  | 1 | 1 | 9.1220 | 0.1720 | 0.0854 | 0.0016 | 5 | 0.4268 | 0.0080 | 0.4349 | 0.00 |
|  | Packaging | Cardboard | 0.0014 |  | 1 | 1 | 0.8840 | 0.1720 | 0.0013 | 0.0002 | 5 | 0.0064 | 0.0012 | 0.0076 | 0.00 |
| Cutimed sorbact gel 7.5cm x 7.5cm | Product | Polyurethane | 0.0017 | 1.56 | 1 | 1 | 0.3000 | 1.0740 | 0.4680 | 0.0018 | 19 | 8.8920 | 0.0343 | 8.9263 | 29.64 |
|  | Packaging | Aluminium | 0.0045 |  | 1 | 1 | 9.1220 | 0.1720 | 0.0406 | 0.0008 | 19 | 0.7714 | 0.0145 | 0.7860 | 0.00 |
|  | Packaging | Cardboard | 0.0025 |  | 1 | 1 | 0.8840 | 0.1720 | 0.0022 | 0.0004 | 19 | 0.0412 | 0.0080 | 0.0493 | 0.00 |
| Flaminal forte gel dressing 15g | Product | Pharmaceuticals | 0.0017 | 4.43 | 1 | 1 | 0.3000 | 1.0740 | 0.0000 | 0.0018 | 10 | 0.0000 | 0.0180 | 0.0180 | 0.00 |
|  | Packaging | LDPE | 0.0045 |  | 1 | 1 | 2.6006 | 0.1720 | 0.0116 | 0.0008 | 10 | 0.1158 | 0.0077 | 0.1234 | 0.00 |
|  | Packaging | Cardboard | 0.0025 |  | 1 | 1 | 0.8840 | 0.1720 | 0.0022 | 0.0004 | 10 | 0.0217 | 0.0042 | 0.0259 | 0.00 |
| Medihoney sheet 5cm x 5cm | Product | Pharmaceuticals | 0.0117 | 1.63 | 1 | 1 | 0.3000 | 1.0740 | 0.4890 | 0.0125 | 10 | 4.8900 | 0.1251 | 5.0151 | 16.30 |
|  | Packaging | Paper | 0.0069 |  | 1 | 1 | 0.9190 | 0.1720 | 0.0063 | 0.0012 | 10 | 0.0634 | 0.0119 | 0.0753 | 0.00 |
| Urgo K-soft 10cm x 4.5m | Product | Polyester | 0.0246 | 0.55 | 1 | 1 | 0.3000 | 1.0740 | 0.1650 | 0.0264 | 16 | 2.6400 | 0.4230 | 3.0630 | 8.80 |
|  | Packaging | LDPE | 0.0019 |  | 1 | 1 | 2.6006 | 0.1720 | 0.0050 | 0.0003 | 16 | 0.0800 | 0.0053 | 0.0853 | 0.00 |
| Urgo K-Lite 10cm x 5.25m | Product | Nilon | 0.0246 | 1.02 | 1 | 1 | 0.3000 | 1.0740 | 0.3060 | 0.0264 | 16 | 4.8960 | 0.4230 | 5.3190 | 16.32 |
|  | Packaging | LDPE | 0.0019 |  | 1 | 1 | 2.6006 | 0.1720 | 0.0050 | 0.0003 | 16 | 0.0800 | 0.0053 | 0.0853 | 0.00 |
| Crepe bandage | Product | Cotton | 0.0051 | 0.55 | 1 | 1 | 0.3000 | 1.0740 | 0.0015 | 0.0055 | 3 | 0.0046 | 0.0165 | 0.0211 | 1.65 |
|  | Packaging | Paper | 0.0022 |  | 1 | 1 | 0.9190 | 0.1720 | 0.0020 | 0.0004 | 3 | 0.0061 | 0.0011 | 0.0072 | 0.00 |
| Profore bandage 10cm x 4.5m | Product | Cotton | 0.0246 | 0.62 | 1 | 1 | 0.3000 | 1.0740 | 0.1860 | 0.0264 | 10 | 1.8600 | 0.2644 | 2.1244 | 6.20 |
|  | Packaging | LDPE | 0.0019 |  | 1 | 1 | 2.6006 | 0.1720 | 0.0050 | 0.0003 | 10 | 0.0500 | 0.0033 | 0.0533 | 0.00 |
| Gauze Pack | Product | Cotton | 0.0051 | 1.71 | 1 | 1 | 0.3000 | 1.0740 | 0.0015 | 0.0055 | 2 | 0.0031 | 0.0110 | 0.0141 | 3.42 |
|  | Packaging | Paper | 0.0022 |  | 1 | 1 | 0.9190 | 0.1720 | 0.0020 | 0.0004 | 2 | 0.0041 | 0.0008 | 0.0048 | 0.00 |
| 0.9% NaCL solution 240ml | Product | Pharmaceuticals | 0.0620 | 2.74 | 1 | 1 | 0.3000 | 0.1720 | 0.8220 | 0.0107 | 6 | 4.9320 | 0.0640 | 4.9960 | 16.44 |
| Normosol irrigation 25ml sachet | Product | Pharmaceuticals | 0.0250 | 0.73 | 1 | 1 | 0.3000 |  | 0.2190 |  | 7 | 1.5330 | 0.0000 | 1.5330 | 5.11 |
|  | Packaging | LDPE | 0.0030 |  | 1 | 1 | 2.6006 | 0.1720 | 0.0078 | 0.0005 | 7 | 0.0546 | 0.0036 | 0.0582 | 0.00 |
| Sodium chloride 0.9% irrigation solution 20ml irripod (Crest medical) - 25 unit dose | Product | Pharmaceuticals |  | 0.73 | 1 | 1 | 0.3000 |  | 0.2190 |  | 2 | 0.4380 | 0.0000 | 0.4380 | 1.46 |
|  | Packaging | LDPE | 0.0060 |  | 1 | 1 | 2.6006 | 0.1720 | 0.0156 | 0.0010 | 2 | 0.0312 | 0.0021 | 0.0333 | 0.00 |
| Dressit Sterile dressing pack | Product | LDPE | 0.0390 | 0.58 | 1 | 1 | 0.3000 | 1.0740 | 0.1740 | 0.0419 | 66 | 11.4840 | 2.7645 | 14.2485 | 38.28 |
|  | Packaging | LDPE | 0.0070 |  | 1 | 1 | 2.6006 | 0.1720 | 0.0182 | 0.0012 | 66 | 1.2015 | 0.0795 | 1.2810 | 0.00 |
|  | Packaging | Paper | 0.0080 |  | 1 | 1 | 0.9190 | 0.1720 | 0.0074 | 0.0014 | 66 | 0.4852 | 0.0908 | 0.5760 | 0.00 |
| Gloves | Product | Rubber | 0.0080 | 0.14 | 1 | 1 | 0.0520 | 1.0740 | 0.0073 | 0.0086 | 66 | 0.4805 | 0.5671 | 1.0476 | 9.24 |

# Resource Use for Patients Without SSI

| Review | | | | | | | | | | | | | | | |
| --- | --- | --- | --- | --- | --- | --- | --- | --- | --- | --- | --- | --- | --- | --- | --- |
| Product | Component | Material | Weight (kg) | Cost (£) | Number of uses | Number of scenarios per use | Emissions factor | Waste stream Emission Factor | Production | Waste | Number | Production total | Waste total | Total emissions | Total Cost (£) |
| Nurse Telephone | Review |  |  | 13.17 | 1 | 1 | 0.1550 |  | 2.0408 |  | 2 | 4.0817 |  | 4.0817 | 26.33 |
| Vascular Telephone Review | Review |  |  | 35.75 | 1 | 1 | 0.1550 |  | 5.5413 |  | 1 | 5.5413 |  | 5.5413 | 35.75 |

| Imaging | | | | | | | | | | | | | | | |
| --- | --- | --- | --- | --- | --- | --- | --- | --- | --- | --- | --- | --- | --- | --- | --- |
| Product | Component | Material | Weight (kg) | Cost (£) | Number of uses | Number of scenarios per use | Emissions factor | Waste stream Emission Factor | Production | Waste | Number | Production total | Waste total | Total emissions | Total Cost (£) |
| USS doppler lower limbs | Review |  |  | 95.00 | 1 | 1 | 0.3000 |  | 0.5300 |  | 6 | 3.1800 |  | 3.1800 | 570.00 |

| Other Consumables | | | | | | | | | | | | | | | |
| --- | --- | --- | --- | --- | --- | --- | --- | --- | --- | --- | --- | --- | --- | --- | --- |
| Product | Component | Material | Weight (kg) | Cost (£) | Number of uses | Number of scenarios per use | Emissions factor | Waste stream Emission Factor | Production | Waste | Number | Production total | Waste total | Total emissions | Total Cost (£) |
| Anticoagulant EDTA (G&S) | Product | LDPE | 0.0200 | 0.07 | 1 | 1 | 0.3000 | 1.0740 | 0.0210 | 0.0215 | 1 | 0.0210 | 0.0215 | 0.0425 | 0.07 |
| Sodium Citrate tube (Coagulation) | Product | LDPE | 0.0080 | 0.20 | 1 | 1 | 0.3000 | 1.0740 | 0.0600 | 0.0086 | 3 | 0.1800 | 0.0258 | 0.2058 | 0.60 |

| Pharmaceuticals | | | | | | | | | | | | | | | |
| --- | --- | --- | --- | --- | --- | --- | --- | --- | --- | --- | --- | --- | --- | --- | --- |
| Product | Component | Material | Weight (kg) | Cost (£) | Number of uses | Number of scenarios per use | Emissions factor | Waste stream Emission Factor | Production | Waste | Number | Production total | Waste total | Total emissions | Total Cost (£) |
| Codeine Phosphate 15mg PO | Product | Pharmaceuticals |  | 0.03 | 1 | 1 | 0.1550 |  | 0.0044 | 0.0000 | 56 | 0.2449 | 0.0000 | 0.2449 | 1.58 |
|  | Packaging | Aluminium | 0.0002 |  | 1 | 1 | 9.1220 | 0.1720 | 0.0015 | 0.0000 | 56 | 0.0838 | 0.0016 | 0.0854 | 0.00 |

| Procedures | | | | | | | | | | | | | | | |
| --- | --- | --- | --- | --- | --- | --- | --- | --- | --- | --- | --- | --- | --- | --- | --- |
| Product | Component | Material | Weight (kg) | Cost (£) | Number of uses | Number of scenarios per use | Emissions factor | Waste stream Emission Factor | Production | Waste | Number | Production total | Waste total | Total emissions | Total Cost (£) |
| 2% chlorhexadine in 70% alcohol skin wipe | Product | Polypropylene | 0.0014 | 0.02 | 1 | 1 | 0.3000 | 1.0740 | 0.0060 | 0.0015 | 1 | 0.0060 | 0.0015 | 0.0075 | 0.02 |
|  | Packaging | Aluminium | 0.0010 |  | 1 | 1 | 9.1220 | 0.1720 | 0.0091 | 0.0002 | 1 | 0.0091 | 0.0002 | 0.0093 | 0.00 |
|  | Packaging | Paper | 0.0003 |  | 1 | 1 | 0.9190 | 0.1720 | 0.0003 | 0.0001 | 1 | 0.0003 | 0.0001 | 0.0003 | 0.00 |
|  | Packaging | LDPE | 0.0003 |  | 1 | 1 | 2.6000 | 0.1720 | 0.0008 | 0.0001 | 1 | 0.0008 | 0.0001 | 0.0008 | 0.00 |
| Syringe 20ml | Product | Polypropylene | 0.0129 | 0.11 | 1 | 1 | 0.3000 | 1.0740 | 0.0330 | 0.0139 | 1 | 0.0330 | 0.0139 | 0.0469 | 0.11 |
|  | Packaging | Paper | 0.0004 |  | 1 | 1 | 0.9190 | 0.1720 | 0.0004 | 0.0001 | 1 | 0.0004 | 0.0001 | 0.0004 | 0.00 |
|  | Packaging | LDPE | 0.0007 |  | 1 | 1 | 2.6000 | 0.1720 | 0.0018 | 0.0001 | 1 | 0.0018 | 0.0001 | 0.0019 | 0.00 |
| Gloves | Product | Rubber | 0.0080 | 0.14 | 1 | 1 | 0.0520 | 1.0740 | 0.0073 | 0.0086 | 16 | 0.1165 | 0.1375 | 0.2540 | 2.24 |

| Dressings | | | | | | | | | | | | | | | |
| --- | --- | --- | --- | --- | --- | --- | --- | --- | --- | --- | --- | --- | --- | --- | --- |
| Product | Component | Material | Weight (kg) | Cost (£) | Number of uses | Number of scenarios per use | Emissions factor | Waste stream Emission Factor | Production | Waste | Number | Production total | Waste total | Total emissions | Total Cost (£) |
| Allevyn GB 7.5cm x 7.5cm | Product | Silicone | 0.0030 | 0.89 | 1 | 1 | 0.3000 | 1.0740 | 0.2670 | 0.0034 | 10 | 2.6700 | 0.0345 | 2.7045 | 8.90 |
|  | Packaging | Paper | 0.0030 |  | 1 | 1 | 0.9190 | 0.1720 | 0.0024 | 0.0005 | 10 | 0.0242 | 0.0045 | 0.0288 | 0.00 |
|  | Packaging | Cardboard | 0.0030 |  | 1 | 1 | 0.8840 | 0.1720 | 0.0032 | 0.0006 | 10 | 0.0316 | 0.0061 | 0.0378 | 0.00 |
| Allevyn GB 10cm x 10cm | Product | Silicone | 0.0120 | 1.29 | 1 | 1 | 0.3000 | 1.0740 | 0.3870 | 0.0125 | 30 | 11.6100 | 0.3754 | 11.9854 | 38.70 |
|  | Packaging | Paper | 0.0070 |  | 1 | 1 | 0.9190 | 0.1720 | 0.0063 | 0.0012 | 30 | 0.1903 | 0.0356 | 0.2259 | 0.00 |
|  | Packaging | Cardboard | 0.0030 |  | 1 | 1 | 0.8840 | 0.1720 | 0.0032 | 0.0006 | 30 | 0.0948 | 0.0184 | 0.1133 | 0.00 |
| Allevyn GB 10cm x 25cm | Product | Silicone | 0.0120 | 2.96 | 1 | 1 | 0.3000 | 1.0740 | 0.8880 | 0.0125 | 10 | 8.8800 | 0.1251 | 9.0051 | 29.60 |
|  | Packaging | Paper | 0.0070 |  | 1 | 1 | 0.9190 | 0.1720 | 0.0063 | 0.0012 | 10 | 0.0634 | 0.0119 | 0.0753 | 0.00 |
|  | Packaging | Cardboard | 0.0040 |  | 1 | 1 | 0.8840 | 0.1720 | 0.0032 | 0.0006 | 10 | 0.0316 | 0.0061 | 0.0378 | 0.00 |
| Allevyn foam 10cm x 20cm | Product | Polyurethane | 0.0180 | 2.62 | 1 | 1 | 0.3000 | 1.0740 | 0.7860 | 0.0193 | 20 | 15.7200 | 0.3866 | 16.1066 | 52.40 |
|  | Packaging | Paper | 0.0060 |  | 1 | 1 | 0.9190 | 0.1720 | 0.0055 | 0.0010 | 20 | 0.1103 | 0.0206 | 0.1309 | 0.00 |
| Softpore 10cm x 15cm | Product | Polyethylene | 0.0017 | 0.09 | 1 | 1 | 0.3000 | 1.0740 | 0.0270 | 0.0018 | 18 | 0.4860 | 0.0325 | 0.5185 | 1.62 |
|  | Packaging | Paper | 0.0009 |  | 1 | 1 | 0.9190 | 0.1720 | 0.0008 | 0.0002 | 1 | 0.0008 | 0.0002 | 0.0010 | 0.00 |
| Inadine 5cm x 5cm | Product | Polyethylene | 0.0019 | 0.37 | 1 | 1 | 0.3000 | 1.0740 | 0.1110 | 0.0021 | 10 | 1.1100 | 0.0207 | 1.1307 | 3.70 |
|  | Packaging | Aluminium | 0.0027 |  | 1 | 1 | 9.1220 | 0.1720 | 0.0244 | 0.0005 | 10 | 0.2444 | 0.0046 | 0.2490 | 0.00 |
|  | Packaging | Cardboard | 0.0007 |  | 1 | 1 | 0.8840 | 0.1720 | 0.0006 | 0.0001 | 10 | 0.0059 | 0.0011 | 0.0071 | 0.00 |
| Inadine 9.5cm x 9.5cm | Product | Polyethylene | 0.0068 | 0.54 | 1 | 1 | 0.3000 | 1.0740 | 0.1620 | 0.0073 | 10 | 1.6200 | 0.0728 | 1.6928 | 5.40 |
|  | Packaging | Aluminium | 0.0032 |  | 1 | 1 | 9.1220 | 0.1720 | 0.0295 | 0.0006 | 10 | 0.2948 | 0.0056 | 0.3004 | 0.00 |
|  | Packaging | Cardboard | 0.0019 |  | 1 | 1 | 0.8840 | 0.1720 | 0.0016 | 0.0003 | 10 | 0.0165 | 0.0032 | 0.0197 | 0.00 |
| Aquacel ribbon 1cm x 45cm | Product | Polyurethane | 0.0210 | 1.31 | 1 | 1 | 0.3000 | 1.0740 | 0.3930 | 0.0226 | 10 | 3.9300 | 0.2255 | 4.1555 | 13.10 |
|  | Packaging | Aluminium | 0.0050 |  | 1 | 1 | 9.1220 | 0.1720 | 0.0456 | 0.0009 | 10 | 0.4561 | 0.0086 | 0.4647 | 0.00 |
| Dressit Sterile dressing pack | Product | LDPE | 0.0390 | 0.58 | 1 | 1 | 0.3000 | 1.0740 | 0.1740 | 0.0419 | 51 | 8.8740 | 2.1362 | 11.0102 | 29.58 |
|  | Packaging | LDPE | 0.0070 |  | 1 | 1 | 2.6006 | 0.1720 | 0.0182 | 0.0012 | 51 | 0.9284 | 0.0614 | 0.9898 | 0.00 |
|  | Packaging | Paper | 0.0080 |  | 1 | 1 | 0.9190 | 0.1720 | 0.0074 | 0.0014 | 51 | 0.3750 | 0.0702 | 0.4451 | 0.00 |
